# ﻿Reclassification of Cybistrinae Sharp, 1880 in the Neotropical Region (Coleoptera, Adephaga, Dytiscidae), with description of new taxa

**DOI:** 10.3897/zookeys.1188.110081

**Published:** 2024-01-08

**Authors:** Kelly B. Miller, Mariano C. Michat, Nelson Ferreira Jr

**Affiliations:** 1 Department of Biology and Museum of Southwestern Biology, University of New Mexico, Albuquerque, NM 87131-0001, USA University of New Mexico Albuquerque United States of America; 2 Instituto de Biodiversidad y Biología Experimental y Aplicada, CONICET-Universidad de Buenos Aires, Buenos Aires, Argentina CONICET-Universidad de Buenos Aires Buenos Aires Argentina; 3 Laboratório de Entomologia, Departamento de Zoologia, Instituto de Biologia, Universidade Federal do Rio de Janeiro, Rio de Janeiro, RJ, Brasi Universidade Federal do Rio de Janeiro Rio de Janeiro Brazil

**Keywords:** Diving beetle, phylogeny, South America, taxonomy, water beetle

## Abstract

The classification of the Neotropical Cybistrinae Sharp, 1880 (Coleoptera: Adephaga: Dytiscidae) is extensively revised based on a phylogenetic analysis of morphological features of the group. A new genus, *Nilssondytes***gen. nov.** is described for a unique new species, *Nilssondytesdiversus***sp. nov.** from Venezuela. The New World genus, *Megadytes* Sharp, 1882, with several subgenera, was found to not be monophyletic. The type species of *Megadytes*, *Dytiscuslatus* Fabricius, 1801 and the species *Cybisterparvus* Trémouilles, 1984 were found to be monophyletic together, and phylogenetically more closely related to *Cybister* Curtis, 1827 than to other species assigned to *Megadytes* sensu stricto, which were found to also be monophyletic. The name *Megadytes* is here restricted to include only *Megadyteslatus* and *Megadytesparvus*. These two species assigned to this newly restricted genus concept are reviewed and diagnosed. A new genus, *Metaxydytes***gen. nov.**, is erected to include all the other species currently assigned to Megadytes sensu stricto. The current subgenus names assigned to Megadytes, *Bifurcitus* Brinck, 1945, *Paramegadytes* Trémouilles & Bachmann, 1980, and *Trifurcitus* Brinck, 1945, are elevated to genus rank since they are variously paraphyletic. The two species assigned to Cybister (Neocybister) Miller, Bergsten & Whiting, 2007, Cybister (Neocybister) festae Griffini, 1895, and Cybister (Neocybister) puncticollis (Brullé, 1837) re reviewed and diagnosed with the former redescribed and its type specimens considered for the first time since its description. Another evidently new species and possible new genus, *Megadytes* species, IR57 (Ribera et al. 2008), from Peru, is also characterized, but not formally treated because of lack of important data for the single, partial specimen. Diagnostic features are illustrated for the entire group.

## Abstract (es)

La clasificación de Cybistrinae Sharp, 1880 neotropicales (Coleoptera: Adephaga: Dytiscidae) se revisa ampliamente con base en un análisis filogenético de características morfológicas del grupo. Un nuevo género, *Nilssondytes***gen. nov.** se describe para la única nueva especie *Nilssondytesdiversus***sp. nov.** de Venezuela. Se encontró que el género, *Megadytes* Sharp, 1882, del Nuevo Mundo y con varios subgéneros, no es monofilético. Se encontró que la especie tipo de *Megadytes*, *Dytiscuslatus* Fabricius, 1801 y la especie *Cybisterparvus* Trémouilles, 1984 forman un grupo monofilético y filogenéticamente más estrechamente relacionado con *Cybister* Curtis, 1827 que con otras especies asignadas a *Megadytes* sensu stricto, que se encontró que también forman un grupo monofilético. El nombre *Megadytes* se restringe aquí para incluir solo a *Megadyteslatus* y *Megadytesparvus*. Se revisan y diagnostican estas dos especies asignadas a este nuevo concepto restringido del género. Un nuevo nombre, *Metaxydytes***gen. nov.**, se erige para incluir a todas las demás especies actualmente asignadas a *Megadytes* sensu stricto. Los nombres subgenéricos actuales asignados a *Megadytes*, *Bifurcitus* Brinck, 1945, *Paramegadytes* Trémouilles y Bachmann, 1980 y *Trifurcitus* Brinck, 1945, se elevan al rango de género, **nuevo estado**, ya que son parafiléticos de diversas formas. Se revisan y diagnostican las dos especies asignadas a Cybister (Neocybister) Miller, Bergsten y Whiting, 2007, Cybister (Neocybister) festae Griffini, 1895 y Cybister (Neocybister) puncticollis, redescribiendo la primera y considerando sus especímenes tipo por primera vez desde su descripción. Otra especie evidentemente nueva y posible nuevo género, *Megadytes*, IR57 (Ribera et al. 2008), de Perú, también se caracteriza, pero no se trata formalmente debido a la falta de datos importantes para el único espécimen parcial. Las características diagnósticas se ilustran para todo el grupo.

## ﻿Introduction

Cybistrinae Sharp, 1880 prior to this paper included seven genera, some with subgenera, from throughout much of the world, but especially in lower latitudes. The classification of the group was phylogenetically revised most recently by Miller et al. (2007). Until now, the New World has included two genera, *Megadytes* Sharp and *Cybister* Curtis. *Megadytes* has included 21 species in four subgenera (Miller and Bergsten 2016; Nilsson and Hájek 2023) mainly from the Neotropical region but also from the southeastern Nearctic. The New World *Cybister* until recently has included three species in C. (Cybister) from North and Central America (Miller 2013), two species of C. (Neocybister) Miller, Bergsten & Whiting from South America and one species, *Cybisterparvus* Trémouilles, from South America, which has remained unplaced with respect to subgenus (Trémouilles and Bachmann 1980; Trémouilles 1984; Miller and Bergsten 2016; Nilsson and Hájek 2023). Remarkably, an overlooked new species of very large-bodied *Cybister* was recently added to Cybister (Cybister) from Mexico, Cybister (Cybister) poblanus Arce-Pérez, Novelo-Gutiérrez & Fery, 2021 supporting the idea that, among water beetles, the larger-bodied groups of species tend to be more overlooked taxonomically (Miller 2013).

Cybistrines in the Neotropical region occur especially in sunny lentic situations with extensive emergent vegetation, although they may be found in many aquatic habitats. Some species can be abundant at certain sites, but many are only rarely collected and are uncommon in collections. Neotropical cybistrines include the largest diving beetles in the world (Hendrich et al. 2019).

Upon the discovery of new taxa and reevaluation of certain described species with some unique character combinations, it became clear that the situation with Cybistrinae in South America is more complicated than the current classification reflects. The goal of this paper is to revise the Neotropical Cybistrinae genus groups and some of the species groups. The largest group of Neotropical species, historically placed in *Megadytes*, is not reviewed here and remains in need of revision with only analysis of the southern species having been made by Trémouilles and Bachmann (1980). The previous paper revising the classification of Cybistrinae of the world by Miller et al. (2007) should be consulted for additional illustrations, details and discussion about the group, and the project presented here supplements that paper specifically.

## ﻿Material and methods

Methods for specimen preparation and examination largely follow Miller et al. (2007) and Miller and Bergsten (2016).

Specimens were examined or are referenced from the following collections:

**DZRJ**Coleção Entomológica Prof. José Alfredo Pinheiro Dutra, Departamento de Zoologia, Universidade Federal do Rio de Janeiro, Rio de Janeiro, Brazil (N. Ferreira Jr);

**KBMC**Kelly B. Miller Collection, University of New Mexico, Albuquerque, NM, USA (K. B. Miller);

**MIZA**Museo del Instituto de Zoología Agrícola Francisco Fernández Yépez, Universidad Central de Venezuela, Maracay, Venezuela (L. Joly);

**MLP**Museo de La Plata, La Plata, Argentina (P. M. Dellapé);

**MNHN**Museum National d’Histoire Naturelle, Paris, France (A. Mantilleri);

**MRSN**Museo Regionale di Scienze Naturali di Torino (Museum of Turin) (F. Giachino);

**MSBA**Museum of Southwestern Biology, Division of Arthropods, University of New Mexico, Albuquerque, NM, USA (K. B. Miller);

**MZLU**Museum of Zoology, University of Lund, Lund, Sweden (J. Ekström);

**MZSP**Museu de Zoologia, Universidade de São Paulo, São Paulo, Brazil (S. A. Casari);

**SEMC**Snow Entomological Collection, University of Kansas, Lawrence, KS, USA (A. E. Z. Short);

**USNM**United States National Museum, Department of Entomology, Washington, DC, USA (L. Chamorro).

Measurements are based on the range of available specimens and/or published values. Measurements were taken either using a standard steel ruler (longer measurements) or an ocular scale on a Zeiss Discovery V8 dissecting microscope at 50× magnification (shorter measurements). Emphasis was placed on measuring the largest and smallest specimens to describe the range of size in a species. Measurements include: (1) total length (TL); (2) greatest width across elytra (GW); (3) greatest pronotal width (PW); (4) greatest width of the head (HW); (5) distance between the eyes (EW); (6) narrowest width of metaventral wing (MV, Fig. 6); and (7) width across lateral portion of metacoxa (MC, Fig. 6). The ratios TL/GW, HW/EW, and MC/MV were also calculated to provide an indication of overall shape, eye size, and relative sizes of morphological features. Published measurements were included for some species if they are outside the range of observed specimens.

Male and female genitalia were dissected using methods similar to Miller (2001), Miller and Bergsten (2014, 2016), and Miller et al. (2007, 2009). Line drawings were created by sketching the structure in pencil using a drawing tube attached to a Zeiss Discovery V8 microscope then scanning and digitizing the sketch, inking, and editing using Adobe Illustrator.

Distribution data are based on examined specimens and published accounts for better-known species. Species historically placed in *Megadytes* have not been revised, and there may be confusion regarding their species identities in publications, so only type localities are referenced for these unrevised groups.

Fresh material suitable for DNA sequence acquisition and analysis was unavailable for many of the new and reinterpreted taxa treated here. Therefore, phylogenetic analysis is based on morphological characters historically used for these groups and a subset of taxa representative of the evident phylogenetic diversity and morphological combinations exhibited by Cybistrinae taxa (e.g., Miller et al. 2007). A few characters are newly analyzed. See below and the Appendix 1 for character discussions.

Numerous characters relevant to Cybistrinae phylogeny are reviewed by Miller et al. (2007) for adults and by Ferreira Jr (2000) and Michat (2006, 2017) for larva and those papers should be consulted for information regarding the morphology analyzed in this paper (see Appendix 1). However, some clarification is required for certain characters important for the classification and reclassification of taxa included here and for Cybistrinae in general. There are also some new characters included in this analysis. These are discussed below and in the Appendix 1. Character coding is included in Suppl. material 1.

One of these problematic characters is the nature of the metatarsal claws (Character (hereafter Char. 25). In certain Australian and Afrotropical Cybistrinae genera (*Austrodytes* Watts, *Onychohydrus* Schaum & White, *Regimbartina* Chatanay, *Spencerhydrus* Sharp & *Sternhydrus* Brinck), the metatarsal claws are unequal in length with the anterior claw shorter than the posterior. But in those species historically placed in *Megadytes* and *Cybister* the claws are characterized by a wider variety of configurations (Figs 15–18, 21–24, 26–29). Some genus groups (e.g., M. (Bifurcitus) Brinck and M. (Trifurcitus) Brinck) have males and females each with equal-length metatarsal claws. Others are sexually dimorphic with either males or both males and females with the posterior claw reduced and shorter than the anterior claw or absent altogether (see Table 1). Because of the complexity of this variation, the claw features are problematic for coding. A single character is analyzed for this with multiple additive states (Char. 25, Figs 15–29).

**Table 1.** Numbers and relative lengths of metatarsal claws in males and females of genera of Cybistrinae.

	Male	Female
*Austrodytes*	2 claws, anterior < posterior	2 claws, anterior < posterior
*Spencerhydrus*	2 claws, anterior < posterior	2 claws, anterior < posterior
*Sternhydrus*	2 claws, anterior < posterior	2 claws, anterior < posterior
*Onychohydrus*	2 claws, anterior < posterior	2 claws, anterior < posterior
*Regimbartina*	2 claws, anterior < posterior	2 claws, anterior < posterior
*Nilssondytes*	2 claws, anterior > posterior	2 claws, anterior > posterior
*Bifurcitus*	2 claws, anterior = posterior	2 claws, anterior = posterior
*Trifurcitus*	2 claws, anterior = posterior	2 claws, anterior = posterior
*Metaxydytes*	2 claws, anterior = posterior	2 claws, anterior > posterior
*Paramegadytes*	2 claws, anterior = posterior	2 claws, anterior > posterior
*Megadytes*	2 claws, anterior > posterior	2 claws, anterior > posterior
*Cybister*	1 claw	Some species with 1 claw, some species with 2 claws, anterior > posterior, few species dimorphic, either 1 claw or 2 claws, if 2, anterior > posterior

Another complicated set of characters includes the male genitalia. The medial margin of male abdominal sternite IX (Char. 31) is either linear (with each medial margin together parallel, Figs 53, 56, 57) or with the medial margins each distinctly emarginate (Figs 51, 52, 54, 55). Emarginate medial margins are characteristic of *Cybister* (Miller et al. 2007) and species here placed in a redefined *Megadytes* (see below). All other Cybistrinae have linear medial margins. Other male genitalic features include the shape of the apex of the male ventral sclerite (Char. 34, see Miller 2001 for discussion of this structure). In South American *Cybister* the apex is distinctly bifid (Figs 31, 34) and in certain species previously in *Megadytes* (see below) it is apicolaterally lobate and finely setose (Figs 42, 43, 45, 47). In other groups it is variable at the species level.

The female reproductive tract (RT) in Cybistrinae is distinctive and requires explanation. In cybistrines there is a single genital opening with an extremely long, heavily muscular vagina (Miller 2001). The spermatheca is elongate and tubular and attached to an enlarged sac-like region at the base of the common oviduct at the end of the vagina (Figs 58, 59, 61–66). There are a pair of structures (possibly gland reservoirs, although sperm have been found in these regions Miller 2001) on the sides of this sac-like region (Char. 36, Figs 58, 59, 61–66). The basic structure is relatively conserved across Cybistrinae with mainly differences in relative lengths of various structures. The gonocoxae are together fused and knifelike for endophytic oviposition (Char. 39, Figs 58–66). The vagina terminates ventrally between the fused gonocoxae with two elongate sclerotized rami (Figs 58–66; Miller 2001). There is some species level variation in the relative sizes and shapes of these structures and whether the rami are smooth (Figs 58–63, 66) or corrugated (Char. 42; Figs 64, 65). The gonocoxosternite exhibits variation in relative size and shape as well (Figs 58–66). The medial margin is distinctly emarginate in Neotropical *Cybister* females (Figs 58, 59) and has a distinctive series of spinous setae in most species previously placed in Megadytes (Megadytes) and Megadytes (Paramegadytes) Trémouilles & Bachmann (Figs 60, 62, 63). Several characters are coded to capture this variation (see Appendix 1).

A particularly problematic set of characters is the subdivisions of antennomeres and maxillary and labial palpomeres in larvae (Chars 45–53). Species of Dytiscinae and Cybistrinae in particular, but other groups as well, have subdivided antennomeres and maxillary and labial palpomeres in various instars giving these structures the appearance of a greater number of segments. Technically they are not additional segments, but are instead subdivisions called articles by Michat et al. (2017). However, homologizing and coding these subdivisions is problematic. For one thing, it can be somewhat challenging determining which of the basic antennomeres and palpomeres are the ones that are subdivided. In addition, it seems likely that these subdivisions may be correlated both within a single larval instar, but also between larval instars and between antennae and palps. That is, it appears that specimens with at least one subdivided antennomere or palpomere have others subdivided, or if subdivided in the antennae, they are also subdivided in the palps making them potentially non-independent. In addition, in some cases, subdivided antennomeres appear to be retained between instars, but not in others (they tend to accumulate between instars). For this analysis, these characters are coded separately, as they are by Michat et al. (2017). An effort was made to maximize information gained by coding these characters but also avoiding overweighting of them. Other phylogenetic characters are discussed more thoroughly in the Appendix 1 and cited references.

The matrix was developed and trees were examined and analyzed using WinClada (Nixon 2002). Characters were analyzed in a parsimony framework using NONA (Goloboff 1995) and the commands “h 10000,” “mu* 400,” and “h/100.” Trees accumulated during this process were further swapped using the command “max*.” Resultant trees were examined under various optimizations and consensus trees were calculated using WinClada (Nixon 2002).

## ﻿Results

### ﻿ Cybistrinae

Taxon classificationAnimaliaColeopteraDytiscidae

Sharp, 1880

B045C7B7-9075-5408-946C-D496EEF7AC9B

Cybistrini Sharp, 1880, as group of ‘Dytiscicomplicati’.

#### Type genus.

*Cybister* Curtis, 1827.

#### Diagnosis and classification.

**Figures 1–8.**
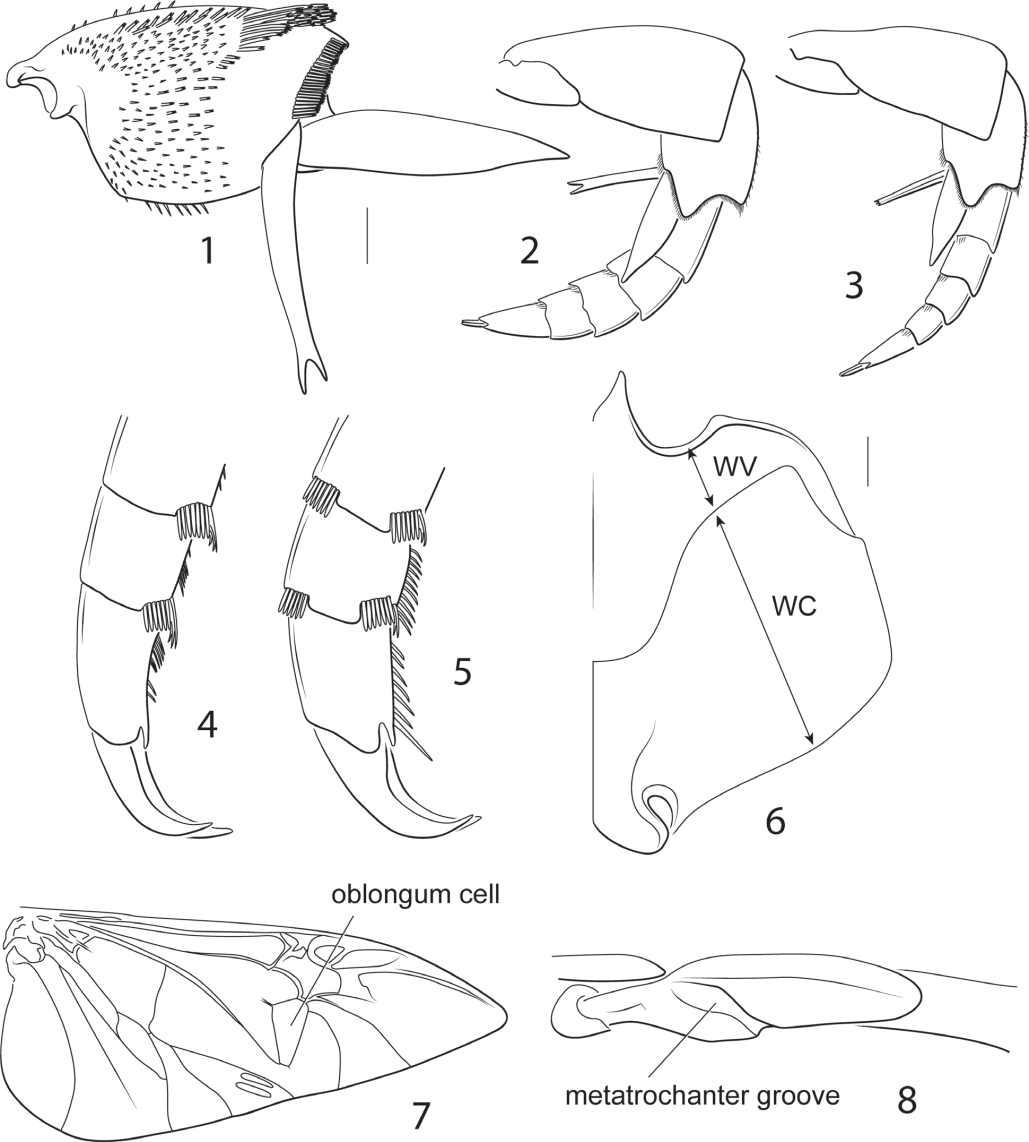
Cybistrinae morphological features **1***Bifurcituslherminieri*, right metatibia, posterior surface **2***Bifurcituslherminieri*, left metaleg, anterior surface **3***Trifurcitusrobustus*, left metaleg, anterior surface **4***Metaxydytesfraternus*, left mesotarsomeres III–V, posterior surfaces **5***Cybistertripunctatus*, left mesotarsomeres III–V, posterior surfaces **6***Megadyteslatus*, left half of metaventrite and left metacoxa (WV = metaventrite wing width, WC = metacoxal width) **7***Cybistertripunctatus*, right metathoracic wing **8***Trifurcitusrobustus*, right metatrochanter, ventral surface. Scale bars: 1.0 mm.

These are large to very large Dytiscidae (length 13.0–47.0 mm). The subfamily is demonstrably monophyletic and is characterized by the following synapomorphies (among others): in adults (Miller et al. 2007; Miller and Bergsten 2014, 2016), (1) the apicoventral elytral setal patch small, composed of a field of short, coarse setae; (2) a large cluster of apically bifid setae present on the posteroapical surface of the metatibia (Fig. 1); (3) the anteroapical metatibial spur acuminate and broader than the posteroapical spur (Figs 1–3); and (4) the oblongum cell sub-triangular (Fig. 7); in larvae (Ferreira Jr 2000; Michat et al. 2017), (5) the anterior margin of the frontoclypeus trilobed (Figs 67–71), (6) antennomeres II and III each subdivided into three articles (in three instars), (7) the premaxillary lobes well developed and projected anteriorly, (8) maxillary palpomere III subdivided into three articles in instars II and III, (9) labial palpomeres I and II each subdivided into two articles in all three instars, (10) a dense row of short spiniform setae in the third basal of the ventral margin of the protarsus (although also characteristic of a number of other diving beetle taxa), (11) protarsus with a ventral row of spines (spinulae, not setae), and meso- and metatarsus each with a row of setae (other dytiscids have spinulae on all tarsi), (12) tergal sclerites reduced to small rectangular plates in abdominal segments I to VI, (13) a subapically located anus, and (14) strongly reduced urogomphi. Male cybistrines have the synapomorphy of protarsomeres I–III broadly laterally expanded into a “palette” that is broader than its medial dimension with a large field of adhesive setae ventrally. Most cybistrines are dark greenish to black, often with a lateral yellow margin along the pronotum and/or the elytron, depending on the species, genus or subgenus. These features with several additional synapomorphies in the female genitalia, larvae, other morphological systems, and DNA sequence data make this group among the most characteristic in Dytiscidae (Nilsson 1988; Ferreira Jr 2000; Miller 2000, 2001; Miller et al. 2007; Miller and Bergsten 2014, 2016; Michat et al. 2017).

Cybistrinae prior to this study included seven genera, several with single or few species and *Megadytes* and *Cybister*, each of which are species rich and include multiple subgenera. The most recent phylogenetic classification of the group was developed by Miller et al. (2007).

The subfamily Cybistrinae has long been associated with Dytiscinae as a tribe of that subfamily and sister to the rest of the clade (e.g., Miller 2000, 2001), but was somewhat reluctantly elevated to subfamily rank by Miller and Bergsten (2014) after they found cybistrines not resolved together with dytiscines. The two clades share an exceptional number of adult and larval features in common, however, and new data and additional taxon sampling may change an understanding of their relationships. Morphological characters supporting monophyly of Dytiscinae and cybistrines are numerous, adults have: (1) the anterior margins of the eyes rounded, not emarginate; (2) the median lobe of the male aedeagus bilaterally symmetrical with a distinct, elongate ventral sclerite; (3) females with a single genital opening in the female reproductive tract for both reception of sperm and oviposition (secondarily within Adephaga); and (4) the female gonocoxae fused together along their dorsal margins, evidently plesiomorphically to facilitate endophytic oviposition although apomorphically this is lost (Miller and Bergsten 2014, 2016). Also, larvae have (among other less clear features): (1) abdominal segments VII–VIII with distinct lateral fringes of natatory setae (present also on abdominal segment VIII in instars II and III of Coptotominae), and (2) the larval antennomeres and maxillary and labial palpomeres subdivided into articles (Ferreira Jr 2000; Michat et al. 2017). Although generally regarded as characteristic of Dytiscinae + Cybistrinae, the subdivision of larval antennomeres and palpomeres in the included taxa is quite variable and likely involves multiple independent characters requiring further investigation to determine homologies within this general condition (see discussion above and character coding scheme below). Subdivision of antennae and palps also occurs (probably homoplasiously) in other diving beetle taxa in different ways.

#### Immature semaphoronts.

Cybistrinae larvae are very characteristic within Dytiscidae (see diagnostic features above). They are often prominent and abundant large predators in systems where they occur. Knowledge of larvae in the group is increasing, but lags behind knowledge of adults, and even lags well behind knowledge of larvae of other diving beetle groups, despite their conspicuousness, although they have been investigated within the context of the phylogeny and taxonomy of Dytiscinae and Cybistrinae (Larson et al. 2000; Alarie et al. 2011; Michat et al. 2017). Table 2 details the state of descriptive knowledge of the morphology of the three instars of each genus group and a key is presented to the known taxa (see below). The pupa of Megadytes (Paramegadytes) glaucus Brullé was described by Crespo (1982). Eggs are unknown for Cybistrinae in general.

**Table 2.** Descriptive knowledge of each larval instar for Cybistrinae. Known instars indicated with “X”.

	Instar			Citations
	I	II	III	
*Austrodytes*	None			
*Bifurcitus*			X	Ferreira Jr (1993, 2000); Michat (2006)
Cybister (Cybister)	X	X	X	Fiori (1949); Watts (1964); Alarie et al. (2011)
Cybister (Megadytoides)	None			
Cybister (Melanectes)	None			
Cybister (Neocybister)	None			
*Megadytes*			X	Ferreira Jr et al. (2006)
*Metaxydytes*	X	X	X	Ferreira Jr (1995); Michat (2010)
*Nilssondytes*	None			
*Onychohydrus*	X	X	X	Watts (1963, 1964); Alarie et al. (2011)
*Paramegadytes*	X	X	X	Crespo (1982); Michat (2006)
*Regimbartina*	None			
*Spencerhydrus*	X	X	X	Michat et al. (2019)
*Sternhydrus*	X	X	X	Watts (1964); Michat et al. (2015)
*Trifurcitus*			X	Ferreira Jr (1995); Michat (2006, 2010)

#### Distribution.

Cybistrinae are found throughout the world, mainly at low latitudes. Most members of the group are tropical, although some occur north to southern Canada and northern Europe and south through temperate South America and Australia and throughout southern Africa.

#### Phylogeny.

Parsimony analysis of the matrix resulted in seven equally parsimonious cladograms (length 102, CI = 68, RI = 93) one of which is shown in Fig. 75 with characters and states optimized on branches. Disagreement among trees is primarily within *Cybister*, but also in relative placement of *Nilssondytes* and *Paramegadytes*. In some solutions, *Paramegadytes* is resolved as sister to a clade of species previously in *Megadytes* (in a new genus described below), and in other solutions is sister to the clade *Megadytes* + *Cybister*. Similarly, *Nilssondytes* is either resolved as sister to a large clade containing a new genus (previously in *Megadytes*), *Paramegadytes*, *Megadytes*, and *Cybister* or is sister to *Megadytes* + *Cybister*. This conflict resulted in a consensus cladogram (Fig. 76) with *Nilssondytes* and *Paramegadytes* in unresolved positions with respect to a new genus (previously in *Megadytes*) and *Megadytes* + *Cybister*.

Cybistrinae and Dytiscinae have historically been regarded as individually monophyletic and together monophyletic (with cybistrines as a tribe within Dytiscinae) based on a large number of adult and larval morphological characters (e.g., Miller 2001, and see above). The most extensive phylogenetic analysis of the family to date by Miller and Bergsten (2014), however, resulted in Cybistrinae and Dytiscinae not together monophyletic, with each of the groups individually monophyletic as historically constituted. In the analysis presented here which is admittedly more limited only to morphological features and fewer taxa, Cybistrinae and Dytiscinae are each monophyletic, and they are together monophyletic (Figs 75, 76).

#### Taxonomic implications of phylogenetic analyses.

An analysis of Cybistrinae and reclassification was presented by Miller et al. (2007) (as Cybistrini in Dytiscinae). Based on that work, Cybistrinae include certain genera characterized by apomorphic features, but also genera characterized by plesiomorphies that do not include any Neotropical species. These are primarily Australian in distribution including *Spencerhydrus* Sharp, 1882, *Austrodytes* Watts, 1978, *Onychohydrus* Schaum & White, 1847, and *Sternhydrus* Brinck, 1945, but also the one Afrotropical species in the genus *Regimbartina* Chatanay, 1911 (Miller et al. 2007). The remaining two genera, *Megadytes* Sharp, 1882 (as historically defined), and its several subgenera, and *Cybister* Curtis, 1827 (also with several subgenera) were found to be together monophyletic based especially (unambiguously) on the presence of an oblique groove across the posterior surface of the metatrochanter (Fig. 8), as well as DNA sequence data, with strong support (Miller et al. 2007). These genera occur in the Neotropical region. *Megadytes* (as then defined) was found to be monophyletic as was *Cybister* (Miller et al. 2007). All South American species of Cybistrinae are evidently part of this clade since they have an oblique, ventral metatrochanteric groove (Miller et al. 2007, and see below).

The analysis presented here is somewhat limited as regards taxon sampling overall, but it expands the Cybistrinae taxa available with morphological data and results largely support previous analyses including; 1) monophyly of Cybistrinae, 2) monophyly of the Australian genera (*Regimbartina* not included here) 2) monophyly of *Cybister* (except *Cybisterparvus*), and 3) monophyly of taxa previously included in *Megadytes* together with *Cybister* (Figs 75, 76; Miller et al. 2007; Miller and Bergsten 2014). However, the addition of newly discovered taxa and poorly known historical taxa with unique new combinations of morphological features resulted in some new phylogenetic relationships. Specifically, *Megadytes*, as historically constituted, is not monophyletic (Figs 75, 76). Previously recognized subgenera of *Megadytes* (several elevated to genus rank, see below) and some of those species historically in Megadytes (Megadytes) (here placed in a new genus, see below) are not monophyletic (Figs 75, 76). An undescribed species from northern South America is ambiguously resolved near these two groups based on a unique combination of features requiring a new genus (Figs 75, 76, see below). In addition, two species (previously *Megadyteslatus* Fabricius and *Cybisterparvus*) with an intermediate character combination between *Megadytes* and *Cybister* are resolved in a monophyletic group between these other two groups requiring generic reclassification, as well (see below).

### ﻿Reclassification of Neotropical Cybistrinae

#### ﻿ Bifurcitus

Taxon classificationAnimaliaColeopteraDytiscidae

Brinck, 1945 stat. nov.

B39BAFF7-362D-5968-AA6A-C3F82FC6FB23

Figs 1 , 2 , 64 , 67

Bifurcitus Brinck, 1945: 8.

##### Type species.

*Cybistergiganteus* Laporte, 1835: 99 by original designation (= *Dytiscuslherminieri* Guérin-Méneville, 1829).

##### Diagnosis.

Within Cybistrinae*Bifurcitus* have (1) the lateral margins of the pronotum and elytra margined with yellow, (2) males and females each with two equal-length metatarsal claws, and (3) the posterior metatibial spur bifid (Figs 1, 2). These are the largest of all diving beetles with adult specimens 36–47 mm in total length (Hendrich et al. 2019). Third instar larvae have (1) the median lobe of the frontoclypeus truncate apically with a tuft of setae (Fig. 67), (2) the median and lateral lobes of the frontoclypeus separated by a wide emargination (Fig. 67), (3) the lateral lobes of the frontoclypeus apically simple (Fig. 67), (4) the lateral lobes of the frontoclypeus obtusely angulate (Fig. 67), and (5) the cephalic capsule relatively short (head length / head width < 1.20).

##### Phylogenetic relationships.

*Bifurcitus* is sister group to the similar *Trifurcitus* (Figs 75, 76; Miller et al. 2007). Both have males and females with equal-length metatarsal claws and the anterior metatibial spur either bifid or trifid (although these two conditions may not be homologous) (Figs 1–3).

##### Discussion.

Although previous evidence suggested that the several subgenera of “*Megadytes*” are monophyletic, monophyly of this group is not supported here based in part on the discovery of undescribed cybistrine species with unique combinations of character states (Figs 75, 76). Given the situation, it seems appropriate to recognize these subgenera at the genus rank including *Bifurcitus*.

There are three currently valid species in *Bifurcitus* which were differentiated and characterized recently by Hendrich et al. (2019).

#### ﻿ Cybister

Taxon classificationAnimaliaColeopteraDytiscidae

Curtis, 1827

AAFD989E-878C-58CD-8615-C552A171AA96

Figs 5 , 7 , 9 , 10 , 15–18 , 30–35 , 51 , 52 , 58 , 59 , 66 , 72

##### Type species.

*Dytiscuslateralis* Fabricius, 1798.

##### Diagnosis.

Within Cybistrinae*Cybister* is characterized by the following: (1) a series of setae present along the posteroventral apical margin of the mesotarsomeres of males and pro- and mesotarsomeres of females (Fig. 5); (2) males with a single metatarsal claw, females with one or two, and if two, then the posterior claw small (some species with females dimorphic, some with a small posterior claw, others with only a single claw) (Figs 15–18); and (3) the medial margin of the lobes of the male abdominal sternite IX emarginate (Figs 51, 52). Larvae of Cybister (Neocybister) are unknown.

##### Distribution.

*Cybister* are found in all major biogeographic regions but are most diverse in the Afrotropical and Oriental regions, mainly in low latitudes. The group is not diverse in the Neotropical region where it is largely replaced in numbers of species and individuals by species previously in Megadytes (Megadytes) (most of these in a newly described genus, see below).

##### Phylogenetic relationships of Neotropical *Cybister*.

*Cybister* is the sister group to *Megadytes* as newly constituted (Figs 54, 76, see below). The Neotropical species of *Cybister* are in the subgenus Cybister (Neocybister) Miller, Bergsten & Whiting, 2007, which is restricted to the New World (Miller et al. 2007). This subgenus was resolved as sister group to all other *Cybister* in the analysis by Miller et al. (2007). It is resolved nested within *Cybister* here based on morphological data (Figs 75, 76), although previously examined molecular data (Miller et al. 2007) are not analyzed here. More investigation is needed. The two South American species are different from other *Cybister* in having (1) females always with a second, rudimentary posterior claw (Figs 15–18), (2) the medial margin of the gonocoxa distinctly emarginate (Figs 58, 59), and (3) the apex of the ventral sclerite of the male median lobe distinctly bifid (Figs 31, 34).

#### ﻿Key to *Cybister* species of the Neotropical region

South of Mexico and Caribbean islands including Cuba and the Bahamas.

**Table:** 

1	Size larger (TL = 26.6–27.6 mm); male median lobe in ventral aspect apically broadly expanded, apex very broad, subtruncate with medial, small point or projection, median lobe broadly expanded medially and apically making lateral margins distinctly sinuate (Fig. 34)	***Cybisterpuncticollis* (Brullé)**
–	Size smaller (TL = 20.3–21.7 mm); male median lobe in ventral aspect apically very slightly expanded, apex truncate without medial small point or projection, median lobe slightly expanded laterally in basal half, but margins not characteristically sinuate (Fig. 31)	***Cybisterfestae* (Griffini)**

#### ﻿ Cybister (Neocybister) festae

Taxon classificationAnimaliaColeopteraDytiscidae

Griffini, 1895

1CBDD198-956B-5EAF-9E6E-24B83A7F006D

Figs 9 , 15 , 16 , 30–32 , 51 , 58 , 72

Cybister festae Griffini, 1895: 1.
Cybister (Meganectes) festae : Brinck 1945: 18.
Cybister (Neocybister) festae : Miller et al. 2007: 54; Nilsson and Hájek 2023: 84.

##### Type locality.

Panama, Darién, Matusagrati Lake (Laguna della Pita).

##### Type specimens.

The syntype specimens are in Museo Regionale di Scienze Naturali di Torino (Museum of Turin, MRSN) (Fig. 72). Images of the specimens were examined (Fig. 72, courtesy of F. Giachino, MRSN). Two specimens are included in the type series, a male and a female (Fig. 72; Griffini 1895). Neither are dissected. The male specimen is here designated as the lectotype to stabilize the nomenclature of the species (see Material examined below; Fig. 72). Although the male is not dissected and the genitalia were not examined, the specimens agree well with the others examined for this study in size, shape, coloration, distribution, and other features.

##### Diagnosis.

This species differs from the other Neotropical *Cybister* species, *C.puncticollis*, in smaller size (TL = 20.3–21.7 mm in *C.festae* vs. 26.6–27.6 mm in *C.puncticollis*) and the shape of the male genitalia. The male median lobe in *C.puncticollis* is apically broadly expanded (Fig. 34) whereas in *C.festae* the median lobe is apically less strongly expanded with lateral margins that are somewhat more parallel-sided (Fig. 31). The specimens examined match the description of *C.festae*, but it is possible that the species illustrated and described here is not the same as *C.festae* since the lectotype (in MRSN) was not dissected.

##### Description.

***Measurements*.**TL = 20.3–21.7 mm, GW = 11.7–13. mm, PW = 8.8–9.2 mm, HW = 5.1–5.5 mm, EW = 2.9–3.0 mm, TL/GW = 1.7–1.8, HW/EW = 1.7–1.8, WC/WV = 4.3–4.4. Body shape oval, widest slightly posteriad of middle; lateral margins broadly curved, continuously curved between pronotum and elytron. Depressed and somewhat flattened in lateral aspect.

**Figures 9–14.**
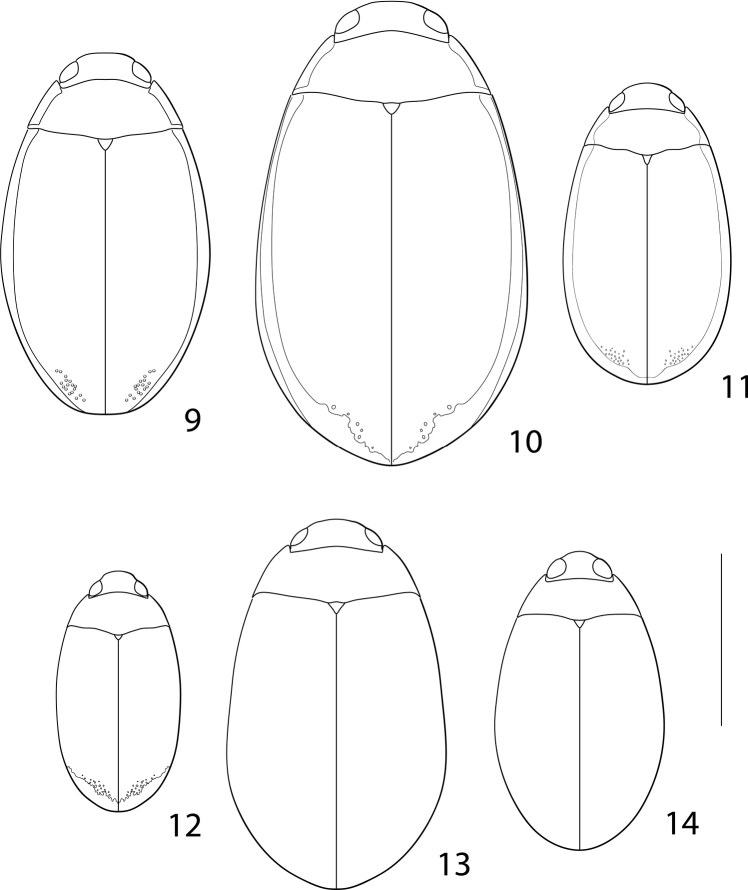
Neotropical Cybistrinae species, habitus **9***Cybisterfestae***10***Cybisterpuncticollis***11***Nilssondytesdiversus***12***Megadytesparvus***13***Megadyteslatus***14** species “IR57” (Ribera et al. 2008). Scale bar: 10.0 mm.

***Coloration*.** Head dark green-black, clypeus and labrum pale yellow. Pronotum dark green-black, with broad lateral yellow marginal band, medial margin of band distinctly delimited, band separated from posterolateral margin by narrow green strip (Fig. 9). Elytron dark green-black with broad lateral yellow marginal band, medial margin of band distinctly delimited, lateral margin of band slightly remote from lateral elytral margin for medial portion of length, extending laterally to margin of elytron anteriorly and posteriorly, apex of yellow band diffusing into coloration of elytron (Fig. 9). Ventral surfaces mostly black except antennae and palpi orange, pro- and mesothoracic legs with basal segments (trochanter and femur) pale orange and apical segments (tibia and tarsus) testaceous, metathoracic legs with mix of testaceous and orange, propleuron and elytral epipleuron pale orange to testaceous, and with small orange maculae laterally on abdominal ventrites IV–VI.

***Sculpture and structure*.** Head broad, anteriorly produced, eyes prominent; dorsal surface evenly covered with exceptionally fine micropunctures but appearing smooth and shiny. Pronotum with lateral margins evenly and shallowly curved; surface of pronotum similar to surface of head in micropunctation; pronotum dorsally evenly curved. Elytron with margins very broadly curved, more strongly curved posteriorly; surface similar to surface of head in micropunctation and surface appearance. Prosternal process anteriorly rounded, surface nearly flat throughout and moderately broad, apex broadly elongate and sharply pointed. Metaventral wing narrow (WC/WV = 4.3–4.4); surface smooth, with extremely fine microsculpture, shiny. Lateral portion of metacoxa large, broad, surface smooth and shiny, with extremely fine microsculpture; metacoxal lines short and strongly curved, well-incised, extending anteriorly less than half distance across metacoxa. Abdominal ventrites smooth, unsculptured.

***Male genitalia*.** Male median lobe in lateral aspect slender throughout length, apically slender, straight, and apically pointed, dorsal sclerite slender, evenly curved basally, apically straight, and slender, (Fig. 30); in ventral aspect broad basally, laterally somewhat constricted medially, apically broadly truncate (Fig. 31). Lateral lobe slender broad basally, apically strongly narrowed and slender, with long dorsal series of setae (Fig. 32).

***Female genitalia* (Fig. 58).** Gonocoxosternite moderately broad, basal portion broadly ovate; gonocoxae together knifelike, evenly convergent to apex; rami smooth, short; vagina elongate; spermatheca elongate, ~ 2/3 length of vagina.

**Figures 15–29.**
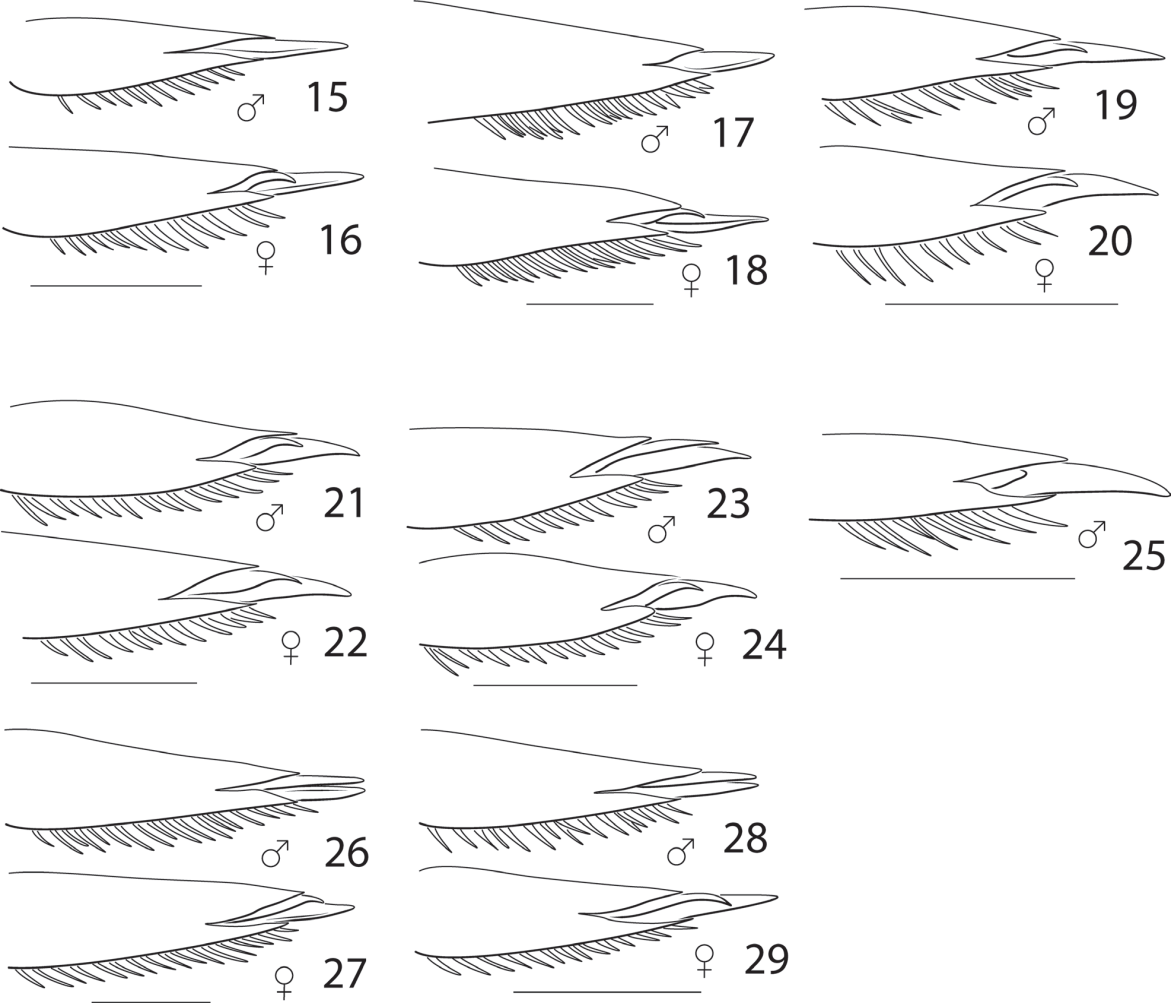
Neotropical Cybistrinae species, metatarsal claws and tarsomere VI of males and females **15, 16***Cybisterfestae***17, 18***Cybisterpuncticollis***19, 20***Nilssondytesdiversus***21, 22***Megadyteslatus***23, 24***Megadytesparvus***25** species “IR57” (Ribera et al. 2008) **26, 27***Paramegadytesglaucus***28, 29***Metaxydytesfraternus*. Scale bars: 1.0 mm.

***Sexual dimorphism*.** Males have a broad protarsal palette with ventral adhesive setae with apical structures in the form of elongate flattened spatulate surfaces; males also have mesotarsomeres with posteroventral fields of setae which are absent in females. Females lack natatory setae along the ventral margins of the metatarsomeres, but these are present in males. Males have a single metatarsal claw (the anterior, Fig. 15), whereas females have a second posterior claw that is ~ 2/3 length of the anterior, curved, and apically sharp (Fig. 16).

***Variation*.** Two specimens were examined, a male and a female. The female has the extent and intensity of greenish-rufous coloration somewhat greater than the male and is larger, but otherwise the two specimens are similar.

##### Distribution.

This species is known from the type locality in the Darién in Panama (Griffini 1895; Brinck 1945) and Venezuela, Apure State (new country record).

##### Material examined.

The lectotype (here designated) in MRSN is labeled, “Laguna d. Pita (Darien) [handwritten]/ Cybisterfestae ♂ Griffini tipo./ Cybisterfestae (tipo) Griffini Darien [green label, black line border, horizontal black lines).” Other than the type specimens (not directly examined), two specimens were examined from Apure State, between La Ye and Bruzual, Venezuela from a roadside lake (7.6443333 -69.3000667) (SEMC, accession numbers: SEMC0846768, SEMC0846766).

#### ﻿ Cybister (Neocybister) puncticollis

Taxon classificationAnimaliaColeopteraDytiscidae

(Brullé, 1837)

3C5E99F8-3F6D-516E-B477-21CBE88F375F

Figs 10 , 17 , 18 , 33–35 , 52 , 59

Dyticus puncticollis Brullé, 1837: 46.
Cybister puncticollis : Aubé 1838: 52.
Trogus puncticollis : Gemminger and Harold 1868: 460.
Megadytes puncticollis : Sharp 1882: 709; van den Branden 1885: 29; Regimbart 1889: 267; 1904: 225; Chatanay 1910: 434; Bruch 1915: 479; Zimmermann 1919: 235; 1920: 256; Wilke 1921: 24; Bruch 1927: 543; Blackwelder 1944: 80; Brinck 1945: 8; Mouchamps 1957: 283; Trémouilles and Bachmann 1980: 105.
Cybister (Meganectes) kemneri Brinck, 1945: 18; Trémouilles and Bachmann 1980: 105.
Cybister (Meganectes) puncticollis : Trémouilles and Bachmann 1980: 105.
Cybister (Neocybister) puncticollis : Miller et al. 2007: 54; Nilsson and Hájek 2023: 84.

##### Type locality.

*Cybisterpuncticollis*: Bolivia, San Miguel. *Cybisterkemneri*: Brazil, La Plata, Amazonas, Rio Autaz.

##### Type specimen.

*Cybisterpuncticollis*, in MNHN (not examined). *Cybisterkemneri*, in MZLU (not examined).

##### Diagnosis.

This species is larger (Fig. 10) than the other Neotropical species in the genus, *C.festae* (Fig. 9) and the male genitalia are distinctly different (Figs 33–35). See above under that species for details about this and additional diagnostic differences between these two species of *Cybister*.

##### Description.

***Measurements*.**TL = 26.6–27.6 mm, GW = 15.0–15.9 mm, PW = 10.8–11.6 mm, HW = 6.5–6.8 mm, EW = 3.7–3.8 mm, TL/GW = 1.7–1.8, HW/EW = 1.7–1.8, WC/WV = 2.9–3.2. Body shape oval, widest slightly posterad of middle; lateral margins broadly curved, continuously curved between pronotum and elytron. Depressed and somewhat flattened in lateral aspect.

***Coloration*.** Head dark green, clypeus and labrum pale yellow. Pronotum dark green, with broad lateral yellow marginal band (Fig. 10), medial margin of band distinctly delimited, band separated from posterolateral margin by narrow green strip (Fig. 10). Elytron dark green with broad lateral yellow marginal band, medial margin of band distinctly delimited, lateral margin of band slightly remote from lateral elytral margin for medial portion of length, extending laterally to margin of elytron anteriorly and posteriorly, apex of yellow band diffusing into coloration of elytron, less distinct (Fig. 10). Ventral surfaces mostly black except antennae and palpi orange, pro- and mesothoracic legs mostly orange, tarsi dark orange, metathoracic legs testaceous, anterodorsal surface of tibia orange, propleuron and elytral epipleuron pale orange to testaceous, and with small orange maculae laterally on abdominal ventrites IV–VI.

***Sculpture and structure*.** Head broad, anteriorly produced, eyes prominent; dorsal surface evenly covered with exceptionally fine micropunctures but smooth and shiny. Pronotum with lateral margins evenly and shallowly curved; surface of pronotum similar to surface of head in micropunctation; pronotum dorsally evenly curved. Elytron with margins very broadly curved, more strongly curved posteriorly; surface similar to surface of head in micropunctation and surface appearance. Prosternal process anteriorly distinctly emarginate medially, remaining surface nearly flat throughout and moderately broad, apex broadly elongate and sharply pointed. Metaventral wing narrow (WC/WV = 2.9–3.2); surface smooth, with extremely fine microsculpture, shiny. Lateral portion of metacoxa large, broad, surface smooth and shiny, with extremely fine microsculpture; metacoxal lines short and curved, extending anteriorly less than half distance across metacoxa. Abdominal ventrites smooth, relatively unsculptured.

***Male genitalia*.** Male median lobe in lateral aspect moderately slender throughout, evenly curved, apically abruptly expanded with a narrowly rounded apex, ventral sclerite slender and evenly curved basally, apically straight and apically pointed (Fig. 33); in dorsal aspect median lobe moderately broad basally, lateral margins broadly sinuate, apex broadly expanded, with apex broadly sub-truncate, medially broadly pointed (Fig. 34). Lateral lobe moderately broad throughout length, evenly curved, apex narrowly rounded, with long series of setae along dorsal margin (Fig. 35).

***Female genitalia*.** Vagina extremely elongate, slender; with enlarged area at base of common oviduct and spermatheca, with enlarged lateral sacs on this enlarged area; spermatheca extremely slender and elongate (Fig. 59); gonocoxae together slender and apically pointed (Fig. 59); gonocoxosternite broad, anterolateral lobe broad, medial margin distinctly, narrowly emarginate, basomedially with field of setae (Fig. 59).

***Sexual dimorphism*.** Males have a broad protarsal palette with ventral adhesive setae with apical structures in the form of elongate flattened spatulate surfaces; males also have mesotarsomeres with posteroventral fields of setae which are absent in females. Females lack natatory setae along the ventral margins of the metatarsomeres, but these are present in males. Males have a single metatarsal claw (the anterior claw, Fig. 17), whereas females have a second, posterior claw that is ~ 1/2 the length of the anterior, is abruptly curved apically and sharply pointed (Fig. 18).

***Variation*.** Specimens vary somewhat in size and coloration, with some specimens more greenish and others darker, less greenish.

##### Distribution.

This species is known from Argentina, Bolivia, Brazil, French Guyana, and Peru. There is also a previously unpublished record from Paraguay.

##### Material examined.

Specimens were examined from Argentina, Bolivia, Peru, and Paraguay.

#### ﻿ Megadytes

Taxon classificationAnimaliaColeopteraDytiscidae

Sharp, 1882

8F1795AC-ACC0-5B4F-A5C5-0BA82BD97C67

Figs 6 , 12 , 13 , 21–24 , 39–47 , 54 , 55 , 60 , 68

Megadytes Sharp, 1882: 701.

##### Type species.

*Megadyteslatus* Fabricius, 1801: 260.

##### Classification.

The concept of the genus presented here differs significantly from previous ones (e.g., Trémouilles and Bachmann 1980; Miller et al. 2007; Miller and Bergsten 2016). The type species of *Megadytes* is *M.latus* Fabricius, 1801, a species which differs in important phylogenetic characters from most other species historically assigned to *Megadytes*. Here the species *M.latus* is placed together with *Megadytesparvus* comb. nov. in *Megadytes*, whereas the other species historically in *Megadytes* are placed in other genera based on the phylogenetic hypothesis developed here (Figs 75, 76). Given the character distribution in this group and the diagnosis of the type species, *M.latus*, this new classification is unavoidably disruptive of the historical concept of the genus (which included all species here placed in the genera *Bifurcitus* stat. nov., *Paramegadytes* new status, and *Trifurcitus* new status, see below) and a new genus described below. The unusual characteristics of *M.parvus* were recognized by Trémouilles (1984) who placed the species in Cybister (Meganectes) Brinck, 1945.

##### Diagnosis.

Within Cybistrinae*Megadytes* are similar to *Cybister* (Figs 51, 52) in having the medial margin of the lobes of the male abdominal sternum IX emarginate (Figs 54, 55), but differ from *Cybister* (Fig. 5) in lacking a series of setae along the posterodorsal apical angle of the mesotarsomeres of males and pro- and mesotarsomeres of females (as in Fig. 4) This places them in an intermediate phylogenetic position between other Cybistrinae and *Cybister* (Figs 75, 76, see below). Males and females both have two metatarsal claws with the posterior claw strongly reduced (Figs 21–24). Third instar larvae (based on *M.latus*) have (1) the median lobe of the frontoclypeus truncate apically with a tuft of setae, (2) the median and lateral lobes of the frontoclypeus separated by a wide emargination (Fig. 68), (3) the lateral lobes of the frontoclypeus apically simple (Fig. 68), (4) the lateral lobes of the frontoclypeus acutely angulate (Fig. 68), and (5) the cephalic capsule relatively long (head length / head width > 1.25).

##### Distribution.

*Megadytes* are found in the Neotropical region. *Megadytesparvus* is known only from the type locality in Bahia State, Brazil, and *M.latus* is known from Brazil, Uruguay, Argentina, French Guiana, and Venezuela.

##### Phylogenetic relationships.

*Megadytes* is sister to *Cybister* (Figs 75, 76) based on both genera with distinctly emarginate medial margins of abdominal sternite IX in males (Figs 51–55). However, *Megadytes* lack a series of setae at the apicodorsal angle of the posterior surface of mesotarsomeres I–IV (and of protarsomeres I–IV of females). This feature is remarkably consistent across the numerous *Cybister* species in the world (Miller et al. 2007).

#### ﻿Key to *Megadytes* species

**Table:** 

1	Size larger (TL = 19.5–23.2 mm), and relatively broader (TL/GW = 1.7–1.8) (Fig. 13); male median lobe in lateral aspect moderately slender, evenly curved throughout along dorsal and ventral margins (Fig. 44)	***Megadyteslatus* (Fabricius)**
–	Size smaller (TL = 13.0–14.6 mm), and relatively narrower (TL/GW = 1.9–2.1) (Fig. 12); male median lobe in lateral aspect broad, subapically with ventral margin (ventral sclerite) abruptly and strongly curved dorsad (Fig. 39)	***Megadytesparvus* (Trémouilles)**

#### ﻿ Megadytes latus

Taxon classificationAnimaliaColeopteraDytiscidae

(Fabricius, 1801)

C3D62DF9-047D-512D-A46A-C42777DC2621

Figs 6 , 13 , 21 , 22 , 44–47 , 54 , 60 , 68

Dytiscus latus Fabricius, 1801: 260.
Trogus latus : Gemminger and Harold 1868:459.
Megadytes latus : Sharp 1882: 706; van den Branden 1885: 110; Chatanay 1910: 435; Bruch 1915: 478; Zimmermann 1919: 235; 1920: 256; Bruch 1927: 543; Guignot 1946: 118; Mouchamps 1957: 282; Trémouilles and Bachmann 1980: 108.
Cybister (Megadytes) latus : Wilke 1921: 248.
Megadytes lata : Blackwelder 1944: 80.
Megadytes (Megadytes) latus : Brinck 1945: 7.

##### Type locality.

South America.

##### Type specimens.

Syntypes in Zoological Museum der Universität Kiel, Germany, not examined.

##### Diagnosis.

This species differs from *M.parvus* in its larger size (TL = 19.5–23.2 mm in *M.latus*, Fig. 13 vs. TL = 13.0–14.6 mm in *M.parvus*, Fig. 12), broader shape (TL/GW = 1.7–1.8 in *M.latus*, Fig. 13, vs. TL/GW = 1.9–2.1 in *M.parvus*, Fig. 12) as well as features of the male genitalia. In *M.latus* the male median lobe is relatively simple and evenly curved in lateral aspect with the apex narrowly rounded to somewhat pointed (Fig. 44). In *M.parvus* the median lobe is very broad in lateral aspect with the dorsal sclerite strongly expanded ventrally and curved with the apex strongly recurved (Fig. 39). The lateral lobe in *M.latus* is very slender apically (Fig. 44), but in *M.parvus* it is relatively broad throughout (Fig. 41).

##### Description.

***Measurements*.**TL = 19.5–23.2 mm, GW = 11.4–13.4 mm, PW = 8.7–10.3 mm, HW = 5.3–6.2 mm, EW = 3.1–4.0 mm, TL/GW = 1.7–1.8, HW/EW = 1.6–1.7, WC/WV = 2.7–3.4. Body shape large and broadly oval, widest slightly posteriad of middle; lateral margins broadly curved, continuously curved between pronotum and elytron. Depressed and somewhat flattened in lateral aspect.

***Coloration*.** Head dark green to dark brown, anteriorly somewhat green-rufous, clypeus pale rufous, labrum pale yellow. Pronotum dark green to dark brown, laterally broadly dark green-rufous. Elytron dark green with broad lateral green-rufous margins in some specimens. Ventral surfaces black, legs dark rufous.

***Sculpture and structure*.** Head broad, anteriorly produced, eyes prominent; dorsal surface evenly covered with exceptionally fine microsculpture and dispersed micropunctures. Pronotum with lateral margins evenly and shallowly curved; surface of pronotum similar to surface of head in microsculpture and micropunctation; pronotum somewhat swollen anteriorly. Elytron with margins very broadly curved, more strongly curved posteriorly; surface similar to surface of head in microsculpture, but with extensive, very fine micropunctation over entire surface. Prosternal process anteriorly rounded, surface flat and moderately broad, apex broadly elongate and sharply pointed. Metaventral wing moderately broad, ~ 1/3 width of lateral portion of metacoxa; surface smooth, without sculpturing. Lateral portion of metacoxa large, broad, surface smooth, without sculpturing; metacoxal lines short and fine, extending anteriorly less than half distance across metacoxa. Abdominal ventrites smooth, unsculptured.

***Male genitalia*.** Male median lobe in lateral aspect moderately slender throughout, evenly curved, apically narrowed, apex narrowly pointed (Fig. 44); in dorsal aspect moderately narrow, apically narrowed to narrowly rounded apex, ventral sclerite slender throughout length, apically abruptly expanded, apex rounded (Fig. 45). Lateral lobe slender throughout length, apically very slender, with long series of setae along dorsal margin (Fig. 46).

***Female genitalia*.** The only female examined has the internal genitalia missing. Externally, the female gonocoxosternite is broad with the medial margin linear; the gonocoxae are together fused and knifelike, broad anteriorly, abruptly constricted subapically and apically linear to narrowly rounded apex (Fig. 60); rami short; other internal structures (vagina, spermatheca, etc.) not observed.

***Sexual dimorphism*.** Males have a broad protarsal palette with ventral adhesive setae with apical structures in the form of elongate flattened structures; males also have mesotarsomeres with posteroventral fields of setae. Females lack natatory setae along the ventral margins of the metatarsomeres, but these are present in males. Males and females each have two metatarsal claws with the posterior shorter, but females have the posterior claw slightly longer than in the male (Figs 21, 22).

***Variation*.** Specimens exhibit variation in size (TL = 19.5–23.2 mm) but are consistent in shape (TL/GW = 1.7–1.8), and male genitalic shape and other features are relatively consistent across the range of the species. There is some variation in coloration with most specimens dark green-black, but a single specimen from the Gran Sabana, Venezuela (MIZA) is dorsally strikingly green. This specimen is also smaller than most (TL = 19.5 mm) and may represent a distinctive regional population or separate species.

##### Distribution.

This species is known from Argentina, Brazil, French Guiana, Uruguay (Sharp 1882; Blackwelder 1944; Mouchamps 1957; Trémouilles and Bachmann 1980; Trémouilles 1989b), and Venezuela (MIZA, new country record).

##### Material examined.

Few specimens of this species exist in collections. Two specimens from Argentina, one from Bolivia (new country record), one from Brazil (KBMC), and a specimen from the Gran Sabana, Venezuela (MIZA) were examined for this study.

#### ﻿ Megadytes parvus

Taxon classificationAnimaliaColeopteraDytiscidae

(Trémouilles, 1984) comb. nov.

B1DF8970-8AE7-5636-92A5-27F84CE8C1F0

Figs 12 , 23 , 24 , 39–43 , 55

Cybister parvus Trémouilles, 1984: 187.

##### Type locality.

Brazil, Bahia State, Santa Rita.

##### Type specimens.

Holotype and nine paratypes in Museu de Zoologia, Universidade de São Paulo, Brazil and two paratypes, one male and one female, in Museo de La Plata, La Plata, Argentina.

##### Diagnosis.

This species differs from the other species in the genus, *M.latus*, in smaller size (TL = 13.0–14.6 mm), narrower shape (TL/GW = 1.9–2.1) (Fig. 12) and features of the male genitalia (Figs 39–43). See above under *M.latus* for details about these differences between the two species.

##### Description.

***Measurements*.**TL = 13.0–14.6 mm, GW = 6.2–7.7 mm, PW = 5.4–5.9 mm, HW = 3.3–3.4 mm, EW = 2.0–2.1 mm, TL/GW = 1.9–2.1, HW/EW = 1.6–1.7, WC/WV = 4.1–4.8. Body shape elongate oval, widest slightly posteriad of middle; lateral margins broadly curved, continuously curved between pronotum and elytron (Fig. 12). Depressed and somewhat flattened in lateral aspect.

***Coloration*.** Head dark green to green-rufous throughout. Pronotum dark green with broad lateral green-rufous margins. Elytron dark green with broad lateral green-rufous margins. Ventral surfaces dark rufous.

***Sculpture and structure*.** Head broad, anteriorly produced, eyes prominent; dorsal surface evenly covered with extremely fine microsculpture and micropunctures. Pronotum with lateral margins evenly and broadly curved; surface similar to surface of head in microsculpture and micropunctation; pronotum somewhat swollen anteriorly. Elytron with margins very broadly curved; surface similar to surface of head in microsculpture, but with extensive, very fine micropunctation. Prosternal process anteriorly rounded, surface flat and broad, apex elongate and sharply pointed. Metaventral wing moderately broad, ~ 1/3 width of lateral portion of metacoxa; surface smooth, without sculpturing. Lateral portion of metacoxa large, broad, surface smooth, without sculpturing; metacoxal lines short, extending anteriorly less than half distance across metacoxa. Abdominal ventrites smooth, unsculptured.

***Male genitalia*.** Male median lobe in lateral aspect broad throughout, subapically somewhat expanded, apically narrowed, apex with multiple small processes (Fig. 39); in dorsal aspect moderately narrow, evenly narrowed apically, apex narrowly lobed, ventral sclerite apically broadly lobed, extending to near apex (Fig. 40). Lateral lobe broad throughout length, evenly curved, apex rounded, with long series of setae along ventral margin (Fig. 41).

***Female genitalia*.** The single female specimen available for examination lacks female genitalia which apparently have been dissected and lost.

***Sexual dimorphism*.** Males have a broad protarsal palette with ventral adhesive setae with apical structures in the form of elongate flattened structures; males also have mesotarsomeres with posteroventral fields of setae. Females lack natatory setae along the ventral margins of the metatarsomeres, but these are present in males. Males and females each have unequal length metatarsal claws with the posterior shorter, but in males the posterior claw is relatively longer than in females, and distinctly, but only somewhat, shorter than the anterior (~ 4/5 of length).

***Variation*.** Two paratype specimens were examined, a male and a female. The female has the extent and intensity of greenish-rufous coloration somewhat greater than the male, but otherwise the two specimens are similar.

##### Distribution.

This species is known only from Santa Rita, Bahia State, Brazil. **I**ndication by Trémouilles (1984) of the locality “Santa Rita” to Goyas State, Brazil is erroneous. In Brazil, there are several locations called Santa Rita, but in all labels of the type materials indicate “Santa Rita BA” in clear reference to Bahia State, Brazil (BA = abbreviation of Bahia) and not to Goiás State (= GO). “Goyaz” is an old spelling of Goiás State.

##### Habitat.

Nothing is known of the habitat of this species.

##### Material examined.

Twelve specimens in MZSP – holotype male and nine paratypes, six males and three females, each specimen labeled, “Santa Rita BA – Brasil IV.1958 E. Dente col. [label with black line border]/ Cybister (Cybister) parvus E. Tremouilles [handwritten] 1990 det. E. R. Tremouilles [label with black line border]/ Cybister (Meganectes) parvus. 1984 Tremouilles [red label, black line border, handwritten]”. Holotype, one paratype female and the other paratypes respectively labeled, “Holotypus [red label, black line border], Holotypus [red label, black line border], Paratypes [label with black line border]”; two paratypes in MLP – one male and one female, each specimen labeled, “Santa Rita BA – Brasil IV.1958 E. Dente col. [label with black line border]/ Cybister (Cybister) parvus E. Tremouilles [handwritten] 1990 det. E. R. Tremouilles [label with black line border]/ PARATYPUS/ MUSEO DE LA PLATA PARATIPO Cybister (Meganectes) parvus. 1984 Tremouilles [red label, black line border, handwritten].”

#### ﻿ Metaxydytes gen. nov.

Taxon classificationAnimaliaColeopteraDytiscidae

4A221B39-BBA5-55B8-8558-DADF58EC87F8

https://zoobank.org/99CCA782-DD1A-4DE2-8D5D-F71909BE0E8F

Figs 4 , 28 , 29 , 57 , 62 , 69

Megadytes sensu auctorum.

##### Type species.

*Megadytesfraternus* Sharp, 1882: 708, by current designation.

##### Diagnosis.

These species have males with equal-length metatarsal claws and females with two claws of unequal length with the posterior claw distinctly reduced (Figs 28, 29). The medial margins of male sternite IX are linear, not emarginate (Fig. 57). This group and *Paramegadytes* are similar in having females with the medial margins of the gonocoxosternite with a series of spinous setae (Fig. 62). From *Paramegadytes* these species differ in being smaller (≤ 24 mm in *Metaxydytes*, compared with ≥ 27 mm in *Paramegadytes*) and having the metasternal wings relatively narrow (WC/WV = 2.5–2.6 in *Metaxydytes*, compared with WC/WV = 1.8–1.9 in *Paramegadytes*). Third instar larvae have; (1) the median lobe of the frontoclypeus truncate apically with a tuft of setae (Fig. 69), (2) the median and lateral lobes of the frontoclypeus separated by a narrow emargination (Fig. 69), and (3) the lateral lobes of the frontoclypeus apically simple and acutely angulate (Fig. 69).

##### Etymology.

*Metaxydytes* is from the Greek *metaxy*, meaning “between,” and *dytes*, meaning “diver,” the root word for many genera of Dytiscidae including in this subfamily. The genus is named to signify its intermediate phylogenetic placement among other genera of Cybistrinae.

##### Phylogenetic relationships.

This genus may be sister group to *Paramegadytes* based especially on the presence of distinctive stiff, spinous setae along the medial margins of the female gonocoxosternite (Miller et al. 2007), although in the analyses presented here the group is ambiguously resolved near *Nilssondytes*, *Paramegadytes* and *Megadytes* + *Cybister* (Figs 75, 76).

##### Discussion.

These species were previous placed in *Megadytes*. The type species of *Megadytes* s. str. is *M.latus* which belongs to a different genus from all other known species previously placed in *Megadytes* (Figs 75, 76) requiring this new name for those species now in *Metaxydytes*. The species of *Metaxydytes* have never been completely revised, although Trémouilles (1989a, b) and Trémouilles and Bachmann (1980) addressed the species in southern South America. The genus is in need of a comprehensive revision.

#### ﻿ Nilssondytes gen. nov.

Taxon classificationAnimaliaColeopteraDytiscidae

20663EA9-E007-5488-B9BA-7699E63FF9FC

https://zoobank.org/DF8AFDB0-B369-4936-B231-84B942B58258

Figs 11 , 19 , 20 , 36–38 , 53 , 61 , 73

##### Type species.

*Nilssondytesdiversus* sp. nov., by current designation.

##### Diagnosis.

From other Cybistrinae this genus differs in having: (1) the metatibial spurs apically simple, (2) metacoxal lines clearly present, (3) the pronotum and elytron with broad, distinct lateral yellow bands along margins (Fig. 11), (4) males and females each with two metatarsal claws, the posterior much reduced in both sexes (Figs 19, 20), (5) the prosternum and prosternal process relatively shallowly but distinctly sulcate, (6) the medial margins of the male sternite IX straight, not emarginate (Fig. 53), (7) no cluster or line of setae at the apicodorsal angle of the posterior surface of the mesotarsomeres, and (8) the ventral surface of the metatrochanter with an oblique, transverse groove. The single species in this genus (described below) is somewhat similar in size, shape and coloration to *Metaxydyteslaevigatus* (Olivier) and may be present among series of that species in collections. *Nilssondytes* differ from *M.laevigatus* in several features (see above) including the presence of yellow lateral elytral margins (Fig. 11) which are absent in *M.laevigatus*. Larvae are unknown.

##### Etymology.

This genus is named *Nilssondytes* from the Latin *dytes* meaning “diver,” and *Nilsson*, after the great diving beetle worker and excellent friend, Anders Nilsson, in honor of his inestimable contribution to the science of diving beetle biology.

##### Phylogenetic relationships.

The single species of *Nilssondytes* is part of the clade that includes species with an oblique metatrochanteric groove, but it has an unresolved position with respect to other genera (Figs 75, 76). The presence of a reduced posterior metatarsal claw in both males and females (Figs 19, 20) with the straight medial margins of the male abdominal sternite IX (Fig. 53) is a unique combination of features within Cybistrinae. Unique among this larger clade is also the sulcate prosternum and prosternal process which is somewhat similar to the Australian genera *Spencerhydrus* and *Sternhydrus*.

#### ﻿ Nilssondytes diversus sp. nov.

Taxon classificationAnimaliaColeopteraDytiscidae

D89DF85F-64A7-51AC-96A3-4B873FC92FF4

https://zoobank.org/3F2BEED6-5AA7-4394-9309-788F3ACFACD1

Figs 11 , 19 , 20 , 36–38 , 53 , 61 , 73 , 74

##### Type locality.

Venezuela, Amazonas State, roadside pond ca. 7 km S Samariapo 5°10.900'N, 67°46.078'W, 95 m elev.

##### Diagnosis.

This is the only species in the genus and is characterized by its diagnostic combination (see above). Typically, species-level features include the shape of the male median lobe which is unique. In ventral aspect the apex is abruptly constricted with the apex narrowly truncate with laterally pointed processes (Fig. 37). In lateral aspect, the median lobe is moderately evenly curved on the dorsal margin, lobe apically abruptly narrowed with the apex elongate and slender, apically narrowly rounded (Fig. 36).

##### Description.

***Measurements*.**TL = 16.7–19.4 mm, GW = 9.6–10.7 mm, PW = 7.0–8.1 mm, HW = 4.2–4.7 mm, EW = 2.7–2.9 mm, TL/GW = 1.7–1.8, HW/EW = 1.6–1.7, WC/WV = 3.1–3.2. Body shape suboval, slightly expanded posteriorly, widest at ~ 3/5 of length; lateral margins evenly, continuously curved between pronotum and elytron. Depressed and somewhat flattened in lateral aspect (Figs 11, 73).

***Coloration*** (Figs 11, 73). Head dark green, anterior clypeal margin yellow, more so laterally, testaceous near eyes. Pronotum dark green with broad lateral yellow margin, posteriorly interrupted and green in one of the four examined specimens, in other specimen yellow extending to posterior angle. Elytron dark green with broad lateral yellow band, separated narrowly from lateral margin, slightly expanded near apex. Ventral surfaces largely black, testaceous on head, basal leg segments and elytral epipleuron.

***Sculpture and structure*.** Head broad, frontoclypeal lines elongate, straight, strongly oblique; anterior clypeal margin broadly, shallowly, and evenly concave; dorsal surface evenly covered with fine microsculpture and micropunctures. Pronotum with lateral margins evenly and broadly curved; surface similar to surface of head in microsculpture and micropunctation. Elytral lateral margin evenly and slightly curved for most of length, apically broadly curved; surface of elytron similar to surface of head in microsculpture and micropunctation. Prosternal process apically rounded, ventral surface distinctly sulcate, apex robust, acutely pointed. Metaventral wing broad, slightly less than 1/3 width of lateral portion of metacoxa (WC/WV = 3.1–3.2); surface smooth, without sculpturing. Lateral portion of metacoxa large, broad, surface smooth, without sculpturing; metacoxal lines short, extending less than half distance across metacoxa. Abdominal ventrites smooth, unsculptured.

***Male genitalia*.** Male median lobe in lateral aspect shallowly curved, apically abruptly narrowed, apex narrowed, slightly curved, apically narrowly rounded, broad medially (Fig. 36). In dorsal aspect broad throughout most of length, apically abruptly narrowed, apex laterally produced, submedially with broad, elongate lobes on each side, ventral sclerite short, apically sharp, acuminate, extending to 3/5 length of median lobe, apex sharply pointed (Fig. 37). Lateral lobe broad in basal half, apically distinctly narrowed, apex narrowly rounded, with series of elongate setae along more than apical half of dorsal margin of lateral lobe (Fig. 38).

***Female genitalia*.** With a single genital opening, vagina elongate, slender, with enlarged, bulbous region at base of common oviduct; spermatheca short, curved, at apex of enlarged region, with soft tissue region on each side of enlarged region (Fig. 61); gonocoxae together broad, apically broadly pointed (Fig. 61); gonocoxosternite broad, with elongate anterolateral lobe, with medial margin sublinear, without conspicuous setae (Fig. 61).

***Sexual dimorphism*.** Males have a characteristic broad protarsal palette with ventral adhesive setae. Males also have mesotarsomeres with clumps of posteroventral setae. Females lack pro- and mesotarsal expansions or adhesive setae. Both males and females have two metatarsal claws with the posterior shorter than the anterior, but females have the posterior somewhat more curved than in males (Figs 19, 20). Females have distinctive microsculpture on the surface of the elytron anteriorly in the form of a field of short striae which is absent in males.

***Variation*.** Five specimens were examined. One specimen has the lateral pronotal yellow band extending to the posterior margin of the pronotum, the others have a narrow dark green separation from the posterior margin.

##### Distribution.

This species is known from few localities in Venezuela along the northwestern margins of the Guiana Shield craton (Fig. 74).

##### Natural history.

The only natural history information available from labels is “roadside pond,” “river margin,” and “rock outcropping.”

##### Etymology.

The species is named from the Latin *diversus*, meaning “different,” in recognition of the different lengths of the metatarsal claws in both males and females (Figs 19, 20).

##### Material examined.

***Holotype***, male labeled, “VENEZUELA: Amazonas State 5°10.900'N, 67°46.078'W, 95 m ca. 7 km S. Samariapo 15.i.2009; leg. Short, Miller, García, Camacho, Joly VZ09-0115-02X: roadside pond/ SM0846115 KUNHM-ENT [barcode label]/ HOLOTYPE: *Nilssondytesdiversus* Miller, Michat and Ferreira-Jr., 2023 [red label with double black line border].” ***Paratypes***, 1 male labeled, “Suapure VENEZ. Caura River 4.20.1900 [handwritten] E.A. Klages.”, 1 female labeled “VENEZUELA: Bolivar State 7°41'23.6"N, 64°1'56.0"W, 134 m ca. 14 km E Rio Aro; 5.viii.2008 leg. A. Short $ M. García AS-08-073; rock outcropping/ SM0829328 KUNMH-ENT [barcode label],” 1 female labeled “VENEZUELA: Guárico State 8°6.226'N, 66°26.228'W, 52 m UCV San Nicolasito Field Station: Rio Aguaro; 10.i.2009 leg. Short, Miller, Joly, García, Camacho; VZ09-0110-01A/ SEMC0852602 KUNHM-ENT,” 1 male labeled “VENEZUELA: Bolivar State 6.58694°N; 67.02912°W Rio Caripito 12.i.2009; leg. Short Miller VZ09-0112-02A: river margin/ SM0844405 KUNHM-ENT [barcode label].” All paratypes with, “…PARATYPE *Nilssondytesdiversus* Miller, Michat and Ferreira-Jr., 2023 [blue label with black line border].”

#### ﻿ Paramegadytes

Taxon classificationAnimaliaColeopteraDytiscidae

Trémouilles & Bachmann, 1980 stat. nov.

EB5DE962-DDCF-57FC-88E9-060CCBFC845D

Figs 26 , 27 , 56 , 63 , 70

Paramegadytes Trémouilles & Bachmann, 1980: 101.

##### Type species.

*Dyticusglaucus* Brullé, 1837: 46 by original designation.

##### Diagnosis.

Like *Metaxydytes* these species have both metatibial spurs apically simple, the medial margins of male abdominal sternite IX straight, and both males and females with two metatarsal claws, males with equal-length claws and females with the posterior claw reduced (Figs 26, 27). Females also share the characteristic of the medial margins of the gonocoxosternite with a series of spinous setae (Fig. 63). The lateral pronotal margin has a diffuse, but distinctive lateral pale band. From *Metaxydytes* these specimens are larger with the metaventrite wings relatively broader (see above under *Metaxydytes* for details of diagnostic comparisons). Third instar larvae have (1) the median lobe of the frontoclypeus truncate apically with a tuft of setae (Fig. 70), (2) the median and lateral lobes of the frontoclypeus separated by a wide emargination (Fig. 70), and (3) the lateral lobes of the frontoclypeus bilobed (Fig. 70).

##### Phylogenetics.

This may be the sister genus to *Metaxydytes* (Figs 75, 76; Miller et al. 2007) although here it is in an unresolved position relative to *Nilssondytes*, *Metaxydytes* and *Megadytes* + *Cybister* (Figs 75, 76). See under *Metaxydytes* for further discussion.

##### Discussion.

There are currently two valid species in this genus, *P.australis* (Germain) and *P.glaucus* (Brullé). Trémouilles and Bachmann (1980) characterized and differentiated them.

#### ﻿ Trifurcitus

Taxon classificationAnimaliaColeopteraDytiscidae

Brinck, 1945 stat. nov.

631B5777-DC5A-5F6C-A278-871946EDC1D5

Figs 3 , 8 , 65 , 71

Trifurcitus Brinck, 1945: 8.

##### Type species.

*Cybisterfallax* Aubé, 1838b: 54, by original designation.

##### Diagnosis.

These are former *Megadytes* species with the anterior metatibial spur apically trifid (Fig. 3). Specimens are very large for diving beetle species (TL = 27–36 mm). They are somewhat similar to *Bifurcitus* specimens. See under that genus for diagnostic comparisons. Larvae are distinctive in having the median lobe of the frontoclypeus sharp apically without an apical tuft of setae.

##### Phylogenetic relationships.

*Trifurcitus* is sister group to *Bifurcitus* (Figs 75, 76). Both males and females have equal-length metatarsal claws and the anterior metatibial spur is either bifid (Figs 1, 2) or trifid (Fig. 3), although these two conditions may possibly not be homologous.

##### Discussion.

See above under *Bifurcitus* for more discussion of these two taxa. Six species are currently recognized. Although they have not been revised thoroughly, most of the species were described or illustrated by Trémouilles (1989a) and Trémouilles and Bachmann (1980).

###### ﻿Other species

#### ﻿“ Megadytes

Taxon classificationAnimaliaColeopteraDytiscidae

species” Ribera et al. 2008

48CC716A-2E36-5B0F-B94A-97BF0A249787

Figs 14 , 25 , 48–50

Megadytes species IR57: Ribera et al. 2008: 25.

##### Discussion.

This single male specimen of a cybistrine from Peru presents some problems. It appears to be an undescribed species based on the male genitalia (Figs 48–50). The specimen was DNA sequenced and analyzed for a project by Ribera et al. (2008) where it was found to be in a group with species then assigned to *Megadytes* (including species of M. (Bifurcitus), M. (Paramegadytes) and M. (Megadytes)). The male specimen currently includes a single metathoracic leg (the other is absent). On it, there are two unequal length metatarsal claws with the posterior short, much shorter than the anterior (Fig. 25), which places it outside the historical diagnosis of *Megadytes* which includes males with equal-length metatarsal claws (although see above). However, the specimen is missing important morphological structures for further interpreting its placement within Cybistrinae including the mesothoracic legs (which are important for examining the posterodorsal series of setae on the mesotarsomeres) and components of the genital capsule (which are important for examining the emargination of the medial margins of abdominal sternite IX). Because of this, the specimen cannot be placed within a known genus. Nor is it reasonable to place it in a new genus or expand the definition of an existing genus to include it given the lack of information about its features. Hopefully, additional specimens will be found to allow this species to be described and placed. The species is described here to the extent possible to allow for future identification and investigation.

**Figures 30–50.**
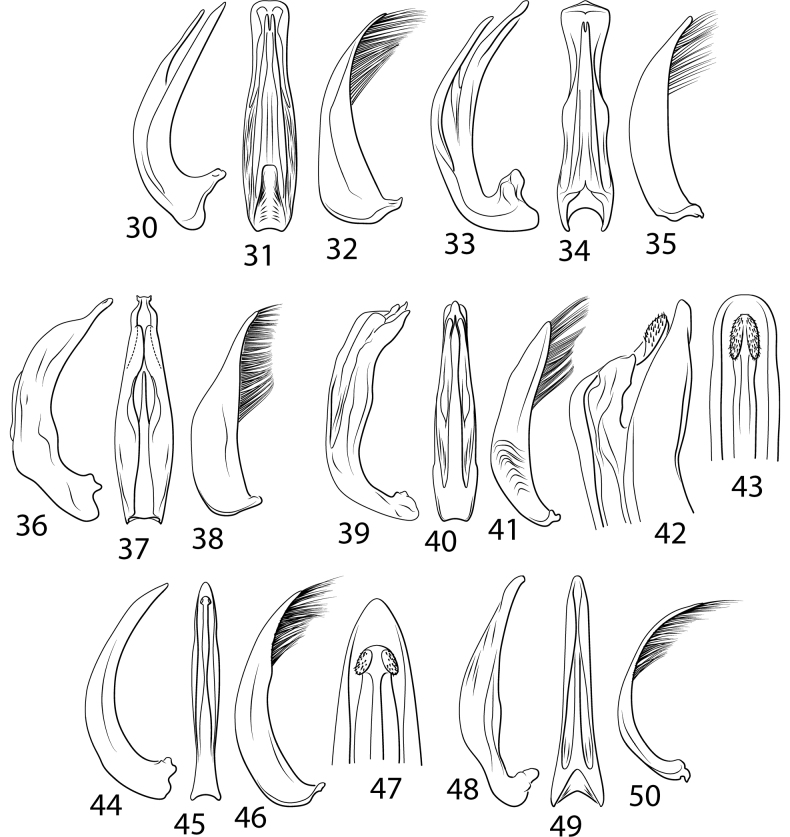
Neotropical Cybistrinae species, male genitalia **30–32***Cybisterfestae*: **30** median lobe, right lateral aspect **31** median lobe, ventral aspect **32** right lateral lobe, right lateral aspect **33–35***Cybisterpuncticollis*: **33** median lobe, right lateral aspect **34** median lobe, ventral aspect **35** right lateral lobe, right lateral aspect **36–38***Nilssondytesdiversus*: **36** median lobe, right lateral aspect **37** median lobe, ventral aspect **38** right lateral lobe, right lateral aspect **39–43***Megadytesparvus*: **39** median lobe, right lateral aspect **40** median lobe, ventral aspect **41** right lateral lobe, right lateral aspect **42** apex of median lobe, right lateral aspect **43** apex of median lobe, ventral aspect **44–47***Megadyteslatus*: **44** median lobe, right lateral aspect **45** median lobe, ventral aspect **46** right lateral lobe, right lateral aspect **47** apex of median lobe, ventral aspect **48–50** species “IR57” (Ribera et al. 2008): **48** median lobe, right lateral aspect **49** median lobe, ventral aspect **50** right lateral lobe, right lateral aspect.

**Figures 51–57.**
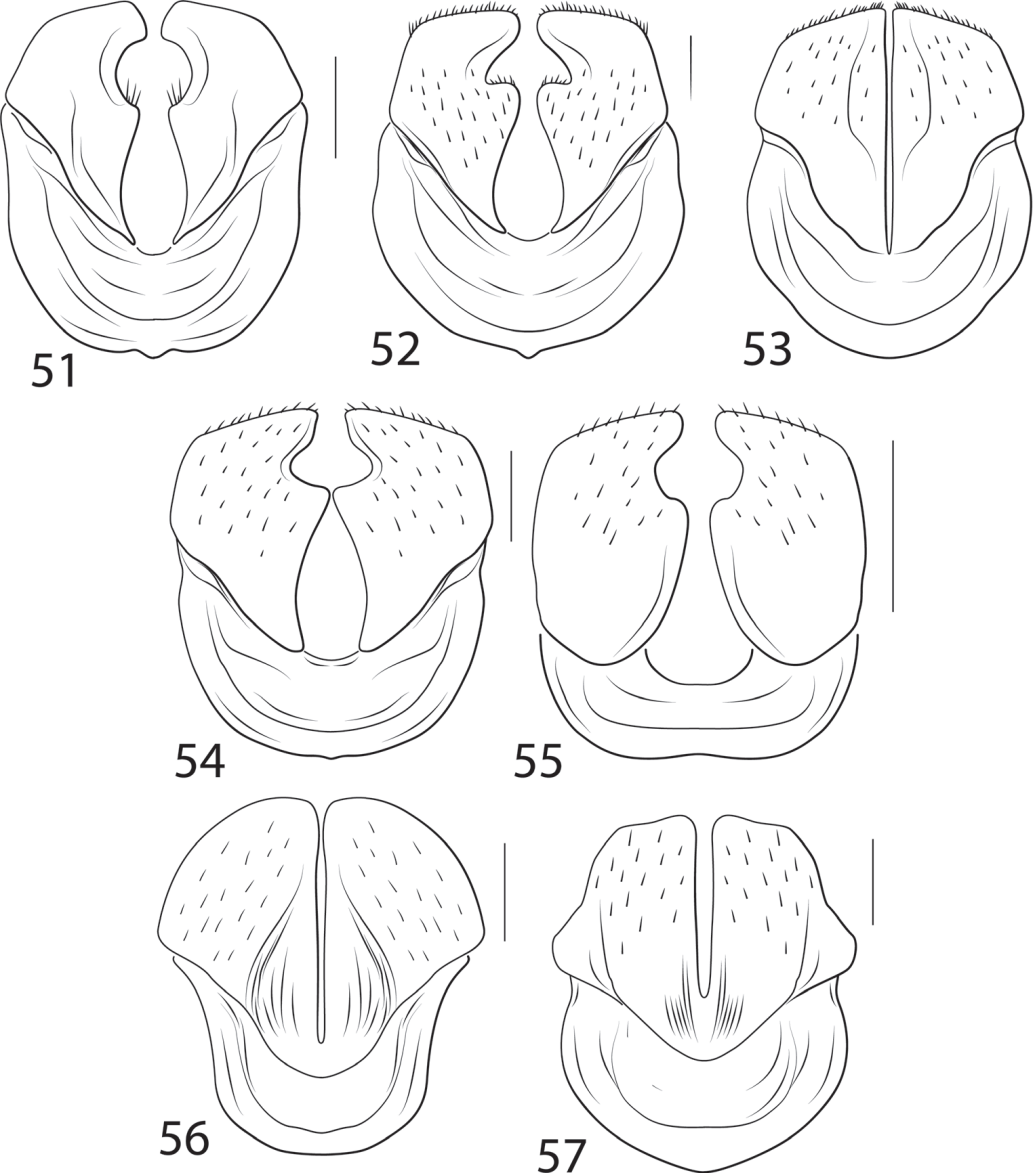
Neotropical Cybistrinae species, male sternite IX, ventral aspect **51***Cybisterfestae***52***Cybisterpuncticollis***53***Nilssondytesdiversus***54***Megadyteslatus***55***Megadytesparvus***56***Paramegadytesglaucus***57***Metaxydytesfraternus*. Scale bars: 1.0 mm.

##### Collection locality.

Peru, Atalapa, Rio Carbon at Rio Madre de Dios, in river, Apr 1999 (Ribera et al. 2008).

##### Description.

***Measurements*.**TL = 17.5 mm, GW = 10.4 mm, PW = 12.8 mm, HW = 4.8 mm, EW = 2.4 mm, TL/GW = 1.7, HW/EW = 2.0, WC/WV = 4.2. Body shape (Fig. 14) broad, expanded posteriorly, widest at ~ 3/5 of length; lateral margins evenly, continuously curved between pronotum and elytron. Depressed and somewhat flattened in lateral aspect.

***Coloration*.** All dorsal surfaces dark reddish brown, without yellow margins on pronotum or elytron. Ventral surfaces entirely dark reddish brown, somewhat more reddish on ventral surfaces of prothorax and pro- and mesothoracic legs.

***Sculpture and structure*.** Head broad; anterior clypeal margin broadly, shallowly and evenly concave; eyes large (HW/EW = 2.0). Dorsal surface shiny and evenly covered with fine micropunctures on head and pronotum, very few sparse micropunctures on elytron. Pronotum with lateral margins evenly and broadly curved. Elytral lateral margin evenly and slightly curved for most of length, apically broadly curved. Prosternal process apically broadly, shallowly concave, ventral surface flat throughout, apex robust, acutely pointed. Metaventral wing narrow, ~ 1/4 width of lateral portion of metacoxa (WC/WV = 4.2); surface smooth, with extremely fine punctation. Lateral portion of metacoxa large, broad, surface smooth, with dispersed, very fine micropunctures; metacoxal lines short, extending only ~ 1/3 distance across metacoxa. Abdominal ventrites smooth, unsculptured.

**Figures 58–61.**
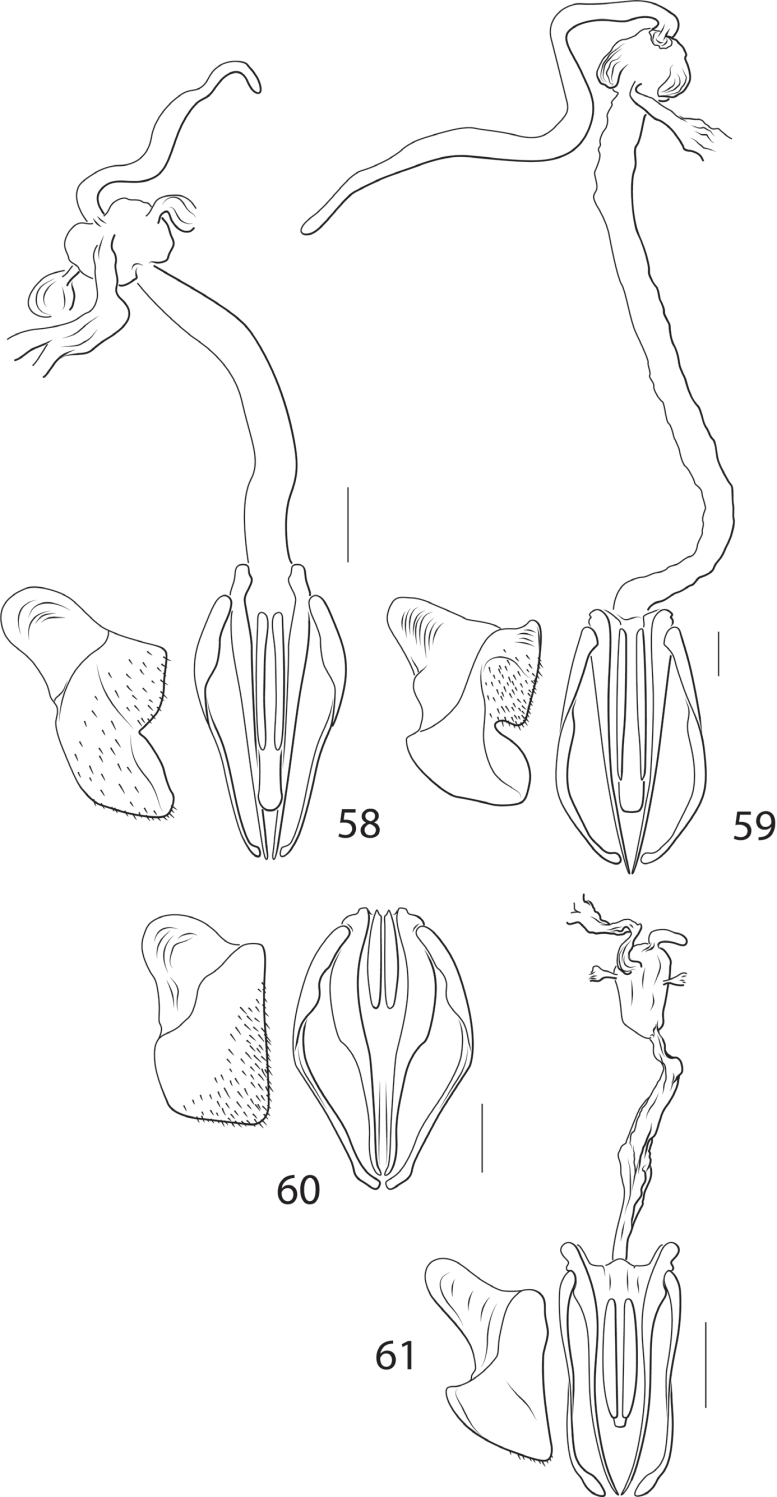
Neotropical Cybistrinae species, female genitalia including left gonocoxosternite, ventral aspect, except Fig. 60 without internal genitalia **58***Cybisterfestae***59***Cybisterpuncticollis***60***Megadyteslatus***61***Nilssondytesdiversus*. Scale bars: 1.0 mm.

**Figures 62–66.**
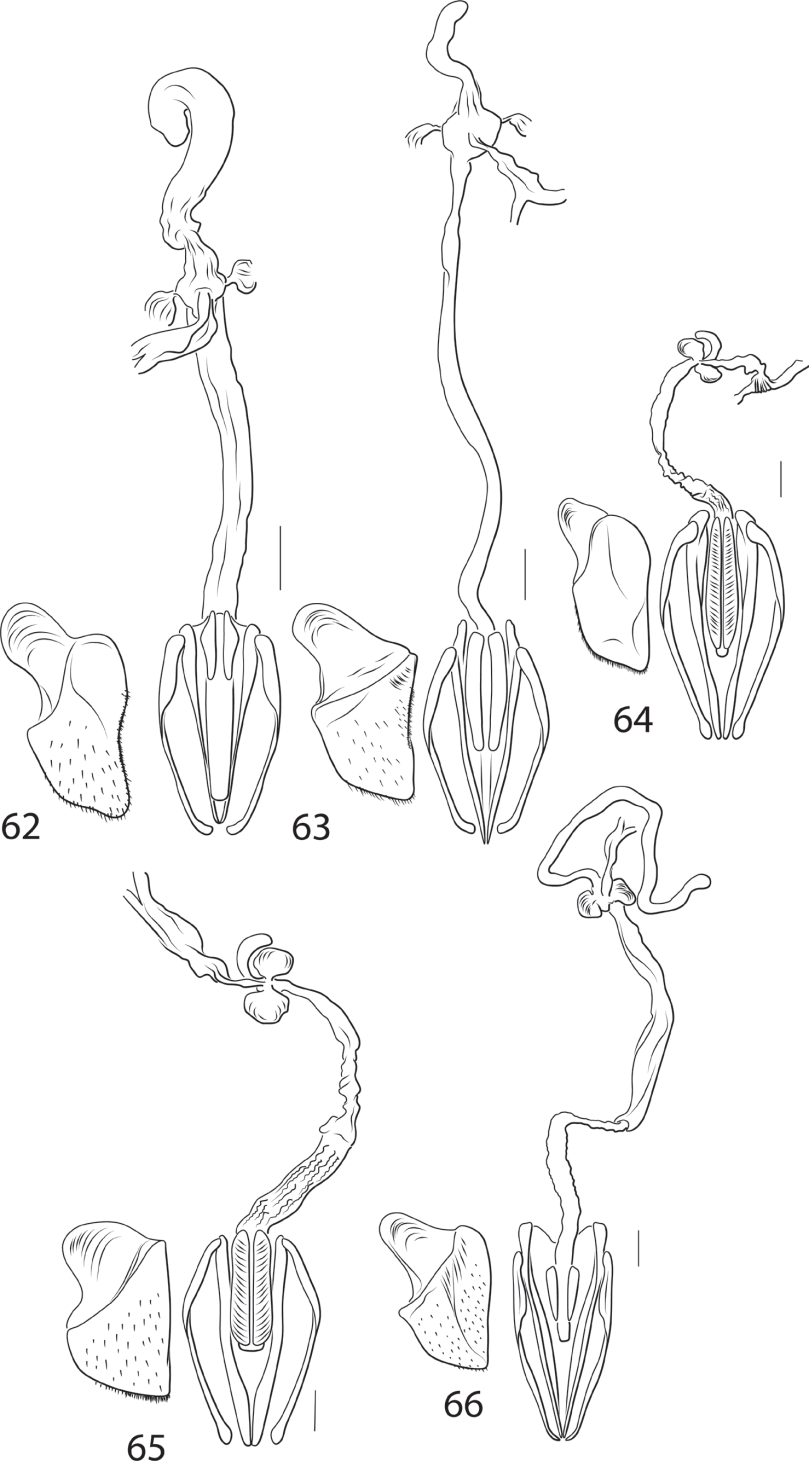
Neotropical Cybistrinae species, female genitalia including left gonocoxosternite, ventral aspect **62***Metaxydytesfraternus***63***Paramegadytesglaucus***64***Bifurcituslherminieri***65***Trifurcitusrobustus***66***Cybisterfimbriolatus*. Scale bars: 1.0 mm.

**Figures 67–71.**
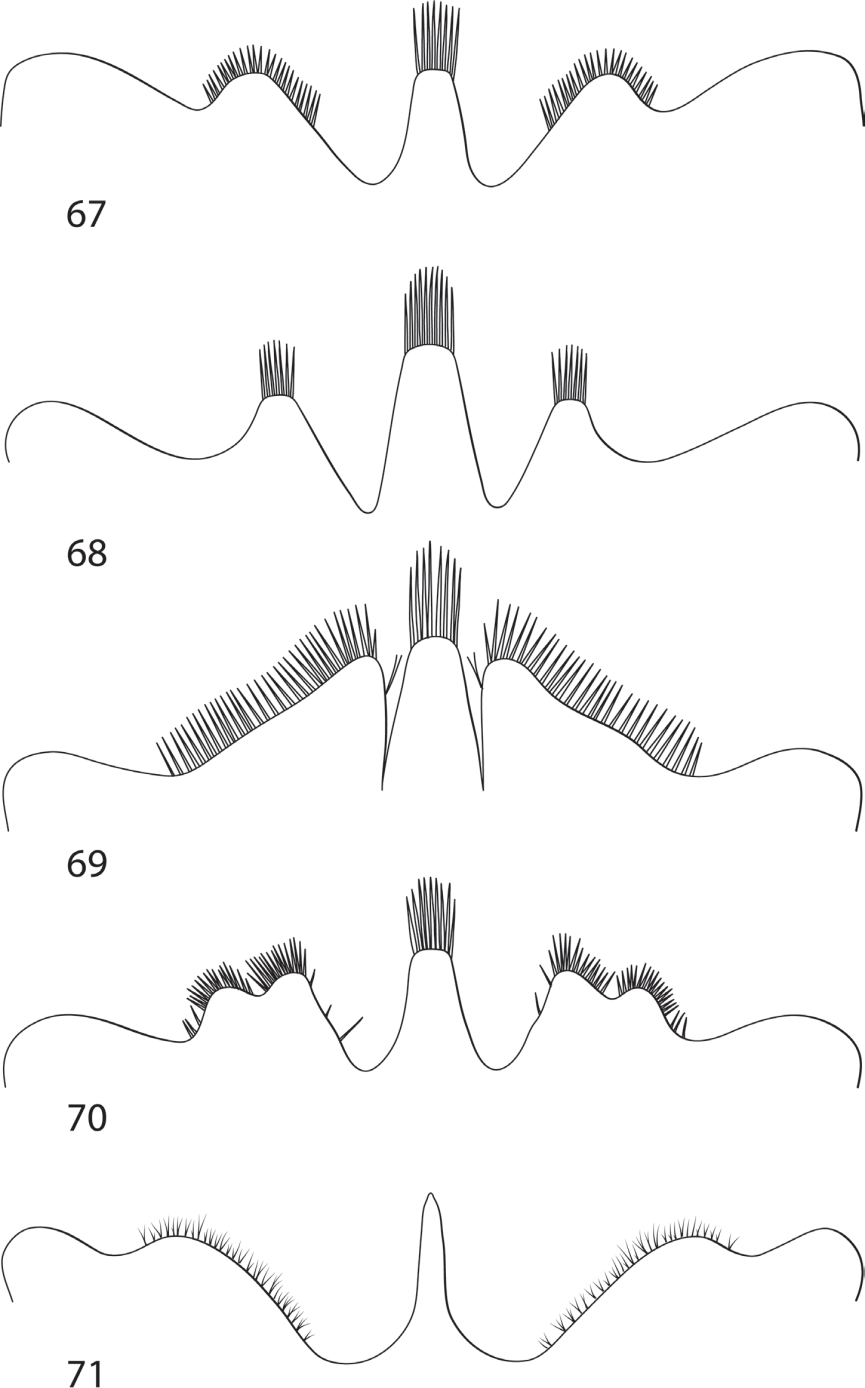
Third instar larva, anterior clypeal margin **67***Bifurcitusmagnus***68***Megadyteslatus***69***Metaxydytescarcharias***70***Paramegadytesglaucus***71***Trifurcitusfallax*.

**Figure 72.**
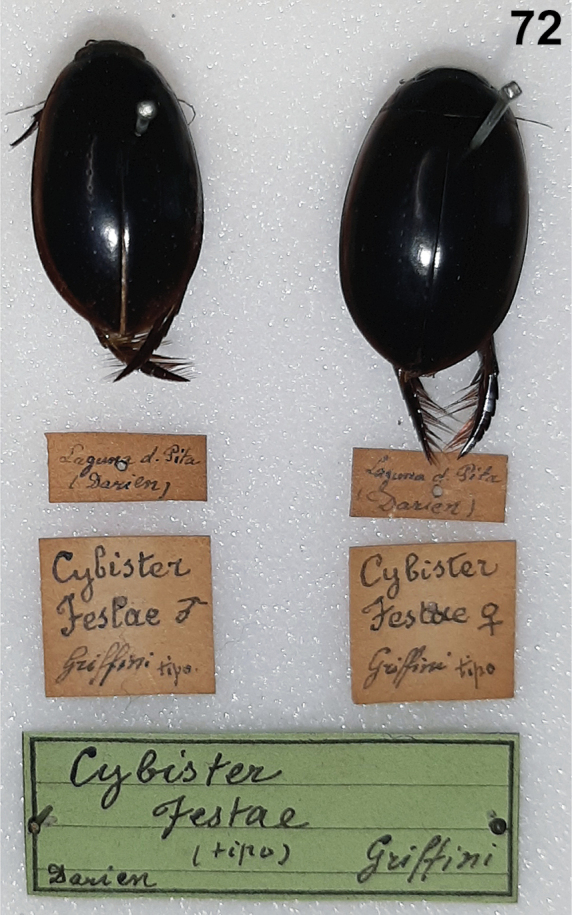
Cybister (Neocybister) festae, lectotype male (left) and paralectotype female (right). Photograph courtesy of F. Giachino, MRSN.

**Figure 73.** *Nilssondytesdiversus*, holotype specimen. Scale bar: 5.0 mm.

**Figure 74.**
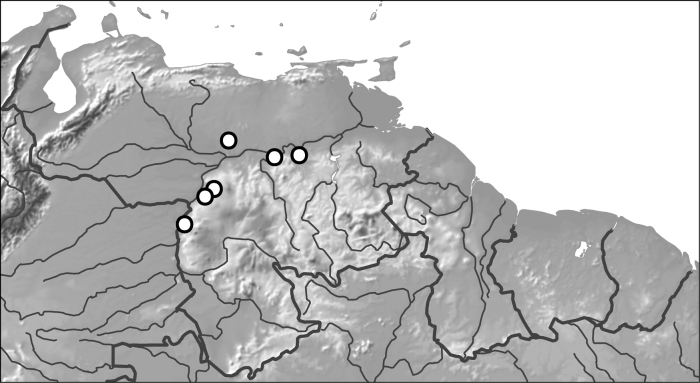
Distribution of *Nilssondytesdiversus* in northern South America.

***Male genitalia*.** Male median lobe in lateral aspect slender, broader submedially and gradually narrowed apically to slightly curved apex, apex bluntly rounded (Fig. 48). In dorsal aspect slender, evenly and gradually narrowed to narrowly rounded apex; dorsal sclerite very slender and elongate (Fig. 49). Lateral lobe extremely slender throughout, broadly curved with long series of long setae along dorsal margin (Fig. 50).

***Female genitalia*.** Females are not known.

***Sexual dimorphism*.** Only a single male was examined. However, this male has a characteristic broad protarsal palette with ventral adhesive setae. Males also have mesotarsomeres with clumps of posteroventral setae. Other typical sexually dimorphic features cannot be compared.

***Variation*.** Only a single specimen was examined.

##### Material examined.

A single male specimen examined labeled, “271297 PERU ZUNGARO COHA 16u:TO [handwritten, somewhat illegible].”

#### ﻿Key to genus groups of Adult Cybistrinae of the world

Adapted from Miller et al. (2007) and Miller and Bergsten (2016) and including revised classification of some groups, see below.

**Table:** 

1	Posterior metatibial spur bi- or trifurcate (e.g., Figs 1, 2); Neotropical	**2**
–	Posterior metatibial spur simple (e.g., Fig. 3); Neotropical and other regions	**3**
2	Posterior metatibial spur bifurcate (Figs 1, 2)	***Bifurcitus* Brinck, stat. nov.**
–	Posterior metatibial spur trifurcate	***Trifurcitus* Brinck, stat. nov.**
3	Prosternal process longitudinally conspicuously sulcate; Australian and Neotropical	**4**
–	Prosternal process without longitudinal sulcus although lateral margins may be variously bordered and anterior portion may be shallowly sulcate or excavated; distribution various	**6**
4	Metacoxal lines absent	***Sternhydrus* Brinck**
–	Metaxocal lines present	**5**
5	Prosternal process longitudinally narrowly and deeply sulcate throughout length; male and female each with metatarsal claws unequal in length, anterior claw shorter than posterior claw	***Spencerhydrus* Sharp**
–	Prosternal process longitudinally broadly sulcate, mainly in anterior half; male and female each with metatarsal claws unequal in length, posterior claw rudimentary and short (Figs 19, 20)	***Nilssondytes* gen. nov.**
6	Metacoxal lines absent; Australian	***Onychohydrus* Schaum**
–	Metacoxal lines present; distribution various	**7**
7	Male with a single metatarsal claw, female either with one claw or with an additional, small posterior rudimentary claw (Figs 15–18); with posteroventral series of setae near apical margin of mesotarsomeres of males and pro- and mesotarsomeres of females (Fig. 5) *Cybister* Curtis)	**8**
–	Male and female with two metatarsal claws, in some cases with posterior claw rudimentary and small (Figs 21–29); without posteroventral series of setae on pro- and mesotarsomeres (Fig. 4)	**11**
8	Elytron without distinct yellow lateral margins; female always with a second, rudimentary posterior metatarsal claw	**9**
–	Elytron with distinct yellow lateral margins; female with either a single metatarsal claw or with a second rudimentary posterior claw	**10**
9	Pronotum with distinct yellow lateral margins	**Cybister (Megadytoides) Brinck**
–	Pronotum without distinct yellow lateral margins	**Cybister (Melanectes) Brinck**
10	Female with a single metatarsal claw or, in few species, dimorphic with some specimens with a second, rudimentary posterior claw; apex of dorsal sclerite of male median lobe various, but not bifid; medial margin of female gonocoxosternite straight or slightly concave; distribution North and Central America, Africa, Eurasia and Australia, absent from most of Neotropical Region except Mexico and certain Caribbean islands	**Cybister (Cybister) Curtis**
–	Female always with two metatarsal claws, posterior claw short and curved (Figs 15–18); apex of dorsal sclerite of male median lobe bifid (Figs 31, 34); medial margin of female gonocoxosternite distinctly emarginate (Figs 58, 59); species from Panama to southern South America	**Cybister (Neocybister) Miller, Bergsten & Whiting**
11	Dorsal surface light green with sparsely distributed, small black dots, laterally without distinct pale margins; central Afrotropical	***Regimbartina* Chatanay**
–	Dorsal surface dark green to green-black without black dots, laterally with or without distinct pale margins; Australian, Nearctic or Neotropical	**12**
12	Prosternal process with distinct lateral carinae; male and female metatarsal claws similar, anterior claw shorter than posterior; dorsal surface dark green with distinct lateral pale margins; Australian	***Austrodytes* Watts**
–	Prosternal process without distinct lateral carinae; male and female claws various, male with either equal-length metatarsal claws or with posterior claw reduced, shorter than anterior, female with two claws, posterior reduced, shorter than anterior (Figs 21–29); dorsal surface dark green to brown or black with or without lateral pale margins; Neotropical or southern Nearctic	**13**
13	Male and female both with two metatarsal claws, anterior claw shorter than posterior (Figs 21–24); male with medial margins of sternite IX emarginate (Figs 54, 55)	***Megadytes* Sharp**
–	Male with two metatarsal claws that are subequal in length, female with two metatarsal claws, with posterior claw shorter, rudimentary (Figs 26–29); male with medial margins of sternite IX straight (Figs 56, 57)	**14**
14	Size large, TL ≥ 27 mm; metasternal wings relatively broad (WC/WV = 1.8–1.9)	***Paramegadytes* Trémouilles & Bachmann, stat. nov.**
–	Size smaller, TL ≤ 24 mm; metasternal wings relatively narrow (WC/WV = 2.5–2.6)	***Metaxydytes* , gen. nov.**

#### ﻿Key to Instar III larvae of Neotropical Cybistrinae

*Nilssondytes* and Cybister (Neocybister) not included (larvae unknown)

**Table:** 

1	Median lobe of frontoclypeus apically sharp, without apical tuft of setae (Fig. 71)	***Trifurcitus* Brinck, stat. nov.**
–	Median lobe of frontoclypeus apically truncate, with apical tuft of setae (Figs 67–70)	**2**
2	Median and lateral lobes of frontoclypeus separated by a narrow emargination (Fig. 69)	***Metaxydytes* , gen. nov.**
–	Median and lateral lobes of frontoclypeus separated by a wide emargination (Figs 67, 68, 70)	**3**
3	Lateral lobes of frontoclypeus bilobed (Fig. 70)	***Paramegadytes* Trémouilles & Bachmann, stat. nov.**
–	Lateral lobes of frontoclypeus with a single lobe (Figs 67, 68)	**4**
4	Lateral lobes of frontoclypeus acute (Fig. 68); cephalic capsule relatively long (ratio head length / head width > 1.25)	***Megadytes* Sharp**
–	Lateral lobes of frontoclypeus obtuse (Fig. 67); cephalic capsule relatively short (ratio head length / head width < 1.20)	***Bifurcitus* Brinck, stat. nov.**

### ﻿List of Neotropical genera and species of Cybistrinae

***Bifurcitus* Brinck, 1945, stat. nov.**

- *Bifurcitusducalis* (Sharp, 1882: 713); Brazil.
- *Bifurcituslherminieri* (Guérin-Méneville, 1829: pl. 8); type locality not given, Guadeloupe by indication.

- = *Cybistergiganteus* Laporte, 1835: 99; Brazil.
- = *Trogusolivieri* Crotch, 1872: 205, by indication to *Dytiscuscostalis* Fabricius sensu Olivier 1795: 9; French Guiana (Cayenne), Suriname.

*Bifurcitusmagnus* (Trémouilles & Bachmann, 1980: 118); Argentina, Santa Fe.

***Cybister* Curtis, 1927**

**Cybister (Neocybister) Miller, Bergsten, & Whiting, 2007**

- *Cybisterfestae* Griffini, 1895: 1; Panama, Darién, Matusagrati Lake (Laguna della Pita).
- *Cybisterpuncticollis* (Brullé, 1837: 46) (*Dytiscus*); Bolivia, San Miguel.

- = *Cybisterkemneri* Brinck, 1945: 18; Brazil, La Plata, Amazonas, Rio Autaz.

***Megadytes* Sharp, 1882**

- *Megadyteslatus* (Fabricius, 1801: 260) (*Dytiscus*); South America.
- *Megadytesparvus* Trémouilles, 1984: 187; Brazil, Bahia State, Santa Rita, comb. nov.

***Metaxydytes* , new genus**

- *Metaxydytescarcharias* (Griffini, 1895: 5) (*Megadytes*); Paraguay, Apa River, Asunción, comb. nov.
- *Metaxydytesecuadorius* (Zimmermann, 1919: 236) (*Megadytes*); Ecuador, Esmeraldas, comb. nov.
- *Metaxydytesflohri* (Sharp, 1882: 709) (*Megadytes*); Mexico, comb. nov. *Metaxydytesfraternus* (Sharp, 1882: 708) (*Megadytes*); Panama, comb. nov.
- *Metaxydytesguayanensis* (Wilke, 1920: 249) (*Cybister*); Guyana, comb. nov.
- *Metaxydytesguignoti* (Mouchamps, 1957: 284) (*Megadytes*); Costa Rica, Bebedero, comb. nov. *Metaxydyteslaevigatus* (Olivier, 1791: 308) (*Dytiscus*); French Guiana (Cayenne), comb. nov. *Metaxydytesmarginithorax* (Perty, 1830: 15) (*Dyticus*); Brazil, comb. nov.
- *Metaxydytessteinheili* (Wehncke, 1876: 359) (*Trogus*); Colombia, Medellín, comb. nov.

***Nilssondytes* , gen. nov.**

- *Nilssondytesdiversus*, sp. nov.; Venezuela, Amazonas State.

***Paramegadytes* Trémouilles & Bachmann, 1980, stat. nov.**

- *Paramegadytesaustralis* (Germain, 1854: 326) (*Cybister*); Chile, Santiago.

- = *Megadytesexpositus* Sharp, 1882: 705; Chile.

- *Paramegadytesglaucus* (Brullé, 1837: pl. 4) (*Dyticus*); Argentina, Buenos Aires; Uruguay, Maldonado, Montevideo.

- = *Cybisteraeneus* Ormancey, 1843: 332; Brazil.
- = *Cybisterbiungulatus* Babington, 1842: 3; Uruguay, Rio de la Plata, Maldonado.

***Trifurcitus* Brinck, 1945, stat. nov.**

- *Trifurcitusaubei* (Wilke, 1920: 245) (*Cybister*), by indication to *Dytiscuscostalis* Fabricius sensu Aubé 1838: 50; French Guiana.
- ssp. *meridionalis* Mouchamps, 1957: 286; Brazil, Amazonas.
- *Trifurcitusfallax* (Aubé, 1838: 54) (*Cybister*); French Guiana (Cayenne).
- *Trifurcitusgravidus* (Sharp, 1882: 712) (*Megadytes*); Brazil, Santa Cruz.
- *Trifurcitusobesus* (Sharp, 1882: 710) (*Megadytes*); Panama.
- *Trifurcitusperplexus* (Sharp, 1882: 711) (*Megadytes*); South America.
- *Trifurcitusrobustus* (Aubé, 1838: 49) (*Cybister*); Brazil.

**Neotropical Cybistrinae incertae sedis with respect to genus**

- ?? *costalis* (Fabricius, 1775: 230) (*Dytiscus*); Suriname.
- ?? *obovatus* (Kirby, 1826: 694) (*Dytiscus*); Brazil.
- “ *Megadytes* species, IR57”, (undescribed species, unknown genus, Ribera et al. 2008).

**Figure 75.**
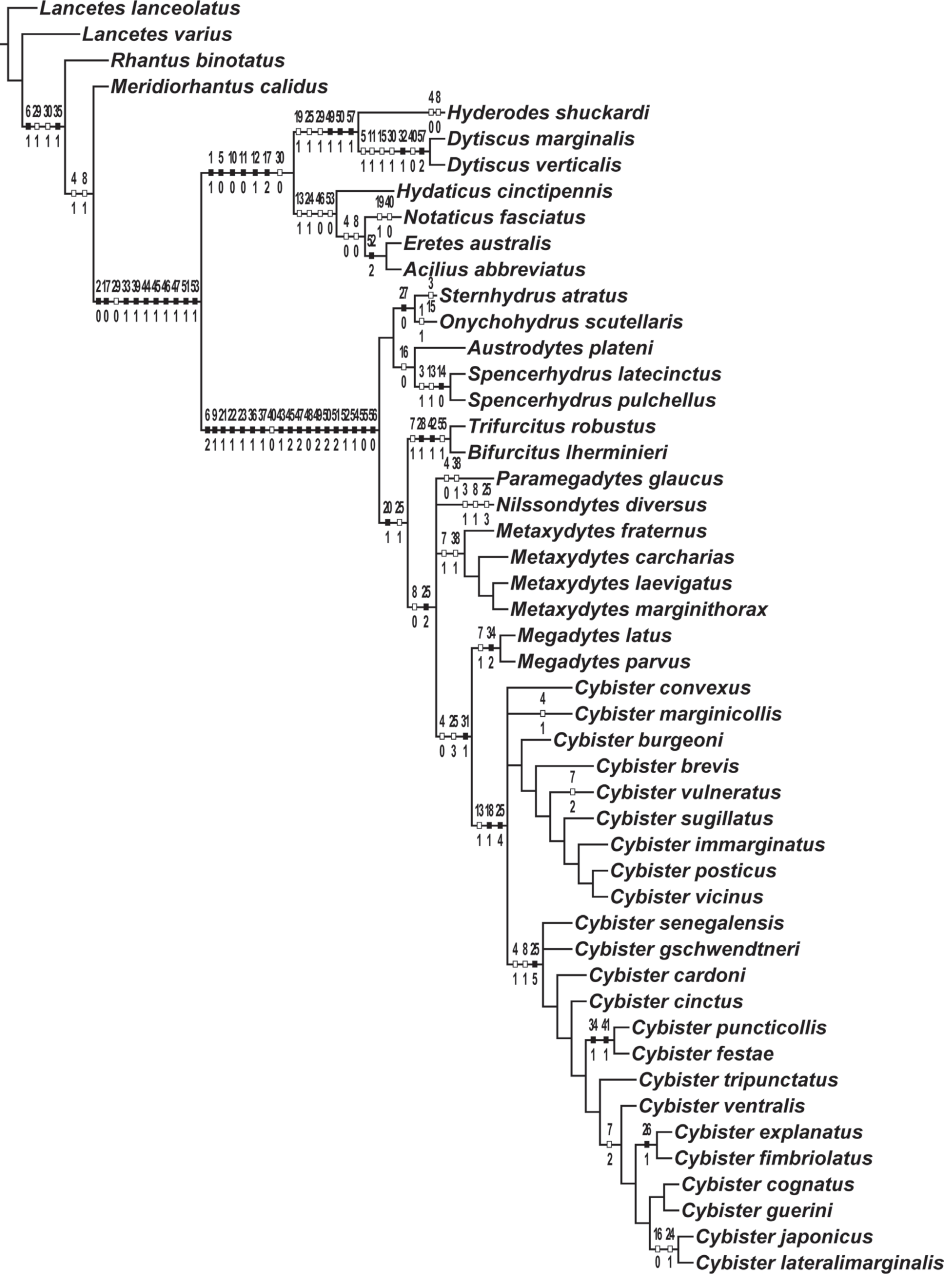
One of seven cladograms derived from parsimony analysis of 57 morphological characters from Cybistrinae and other Dytiscidae (len = 105, CI = 68, RI = 92) with characters mapped using ‘fast’ optimization in WinClada. Black hash marks indicate unambiguous changes, white hash marks indicate homoplasious changes or reversals. Numbers above hash marks are character numbers, those below hash marks are derived state numbers.

**Figure 76.**
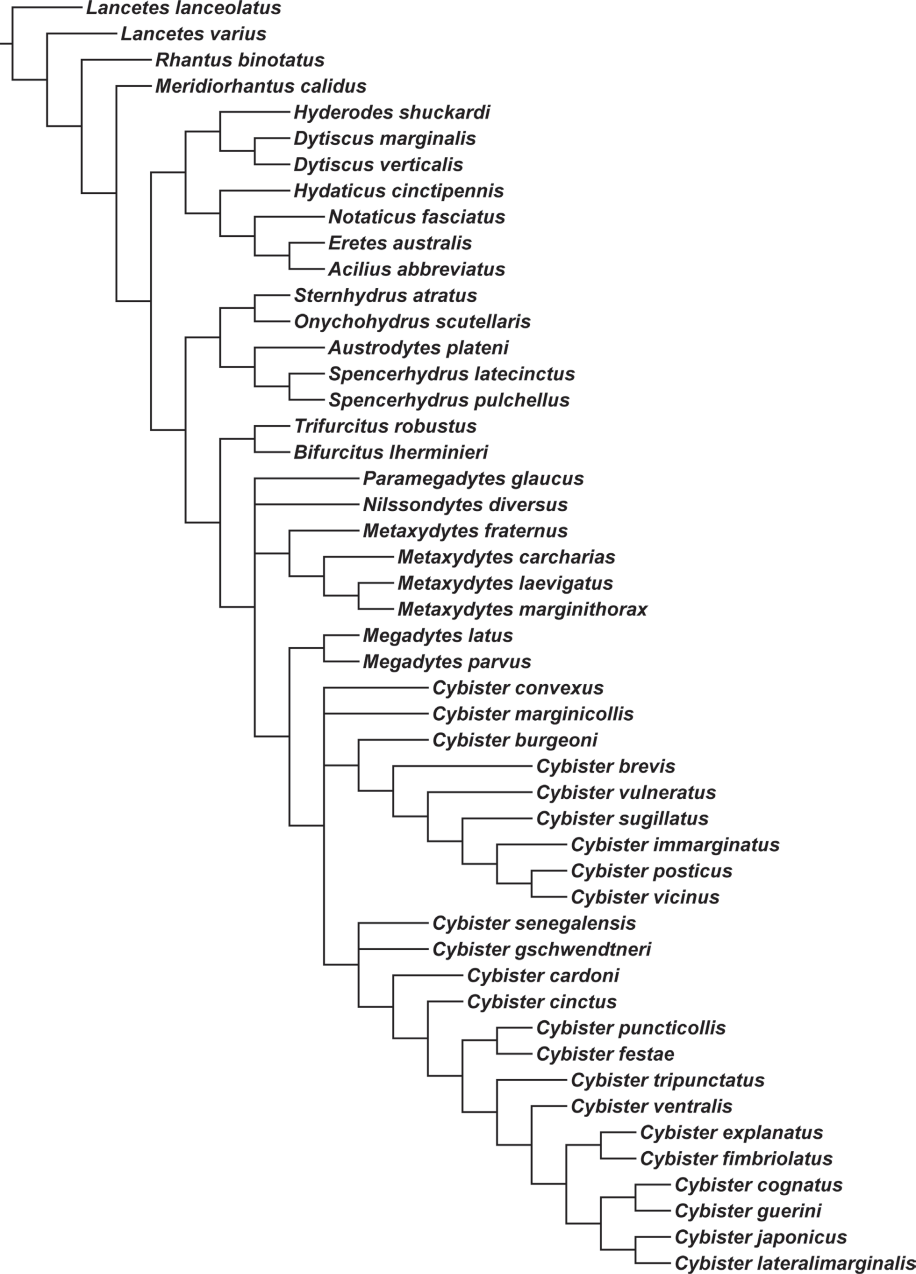
Consensus cladogram of seven equally parsimonious cladograms from parsimony analysis of 57 morphological characters from Cybistrinae and other Dytiscidae morphology.

## Supplementary Material

XML Treatment for Cybistrinae

XML Treatment for Bifurcitus

XML Treatment for Cybister

XML Treatment for Cybister (Neocybister) festae

XML Treatment for Cybister (Neocybister) puncticollis

XML Treatment for Megadytes

XML Treatment for Megadytes latus

XML Treatment for Megadytes parvus

XML Treatment for Metaxydytes

XML Treatment for Nilssondytes

XML Treatment for Nilssondytes diversus

XML Treatment for Paramegadytes

XML Treatment for Trifurcitus

XML Treatment for Megadytes

## ﻿Additional information

### Author ORCIDs

Kelly B. Miller https://orcid.org/0009-0004-1093-4066

Mariano C. Michat https://orcid.org/0000-0002-1962-7976

Nelson Ferreira Jr https://orcid.org/0000-0002-5932-7695

### Data availability

All of the data that support the findings of this study are available in the main text or Supplementary Information.

## ﻿Appendix 1

Morphological characters used in phylogenetic analysis. Citations are provided for previous use of the characters in phylogenetic analyses. These should be consulted for more detailed descriptions and review of these characters.

**Adult**

***Head***

1. Setae on mandible; (0) discontinuous line, (1) continuous line (Char. 1, Miller 2000).
2. Eyes; (0) anteriorly emarginate, (1) anteriorly rounded (Char. 2, Miller 2000).

***Thorax***

1. Prosternal process; (0) not sulcate, (1) sulcate (Char. 48, Miller 2001).
2. Lateral marginal yellow band on pronotum; (0) absent (Figs 12–14), (1) present (Figs 9–11) (Char. 4, Miller et al. 2007).
3. Apicoventral setal patch on elytron; (0) absent, (1) present (Char. 8, Miller 2000).
4. Apicoventral setal patch on elytron; (0) line of stiff setae only along apical margin, (1) large field of fine setae, (2) large field of stiff setae (Char. 9, Miller 2000). Coded as “?” for taxa without an apicoventral setal patch (Char. 5).
5. Sexual sculpture on female elytron; (0) without short striae, (1) with short, longitudinal striae on elytra (2) with extensive anastomozing lines (Char. 7, Miller 2003).
6. Lateral marginal yellow band on elytron; (0) absent (Figs 12–14), (1) present (Figs 9–11) (Char. 9, Miller et al. 2007).
7. Oblongum cell on metathoracic wing; (0) oval shaped, (1) subtriangular (Fig. 7) (Char. 10, Miller et al. 2007).

***Legs***

1. Male anterior protibial spur; (0) absent, (1) present (Char. 11, Miller 2000).
2. Male posterior protibial spur; (0) absent, (1) present (Char. 12, Miller 2000).
3. Ventral protarsal adhesive setae on male; (0) apically with elongate, thin, flattened structures, (1) apically rounded, sucker-shaped (Char. 13, Miller 2000).
4. Posteroapical marginal setae on mesotibia; (0) absent medially, (1) present across entire margin (Char. 15, Miller 2000).
5. Posterodorsal series of setae on mesotibia; (0) apically simple, (1) apically bifid (Char. 17, Miller 2000).
6. Posteroventral series of setae on mesotibia; (0) apically simple, (1) apically bifid (Char. 18, Miller 2000).
7. Ventral adhesive setae on male mesotarsomeres; (0) absent, (1) present (Char. 22, Miller 2000).
8. Ventral adhesive setae on male mesotarsomeres; (0) dense field of apically simple setae, (1) apically with elongate, thin, flattened structures, (2) setae apically sucker-shaped (Char. 23, Miller 2000). Coded as “?” for taxa without ventral adhesive setae on male mesotarsomeres (Char. 15).
9. Line of setae at posterodorsal apical angle on mesotarsomeres I–IV (and protarsomeres I–IV of female); (0) absent, (1) present. Note that this series of setae was erroneously described as being “posteroventral” in position by Miller et al. (2007). All cybistrines have posteroventral series of setae, but only members of *Cybister* have a posterodorsal series of setae. This error was pointed out and corrected by Arce-Pérez et al. (2021).
10. Metacoxal processes: (0) not concave laterally; (1) concave laterally (Char. 6, Miller 2000).
11. Oblique groove across posterior surface of metatrochanter; (0) absent, (1) present (Char. 24, Miller 2000).
12. Natatory setae on dorsal margin of metafemur; (0) absent, (1) present (Char. 27, Miller 2000).
13. Anterior metatibial spur; (0) similar to posterior spur, unmodified, (1) apically acuminate, much broader than posterior spur (Figs 1–3) (Char. 31, Miller 2000).
14. Posterodorsal series of setae on metatibia; (0) a linear series, (1) a cluster (Fig. 1) (Char. 29, Miller 2000).
15. Natatory setae on ventral margin of metatarsomeres; (0) absent on females, present on males, (1) present in both sexes (Char. 36, Miller 2000).
16. Metatarsal claws; (0) male and female each with two claws, anterior shorter than posterior, (1) male and female with two claws, each the same length, (2) male and female with two claws, male with each the same length, female with posterior shorter than anterior (Figs 26–29), (3) male and female each with two claws, posterior shorter than anterior (Figs 19–25), (4) male with a single claw, female with two claws, posterior shorter than anterior (Figs 15–18), (5) male and female each with a single claw (Char. 35, Miller 2000).
17. Male stridulatory device using metacoxa and metatrochanter: (0) absent; (1) present (Char. 26, Miller et al. 2007).
18. Metacoxal lines; (0) absent, (1) present (Char. 27, Miller et al. 2007).
19. Anterior metatibial spur; (0) simple, (1) bifid or trifid (Figs 1–3). It seems likely that a spur that is apically branched is homologous whether it is bifid or trifid.

***Abdomen***

1. Transverse carinae on dorsolateral surface of abdominal ventrite II; (0) absent, (1) present (Char. 59, Miller 2001).

***Male genitalia***

1. Series of long setae on dorsal margin of lateral lobe; (0) absent, (1) present (Figs 32, 35, 38, 41, 46, 50) (Char. 38, Miller 2000).
2. Medial margin of male abdominal ventrite IX; (0) linear (Figs 53, 56, 57), (1) emarginate (Figs 51, 52, 54, 55).
3. Setae on dorsal margin of median lobe; (0) absent, (1) present (Char. 54, Miller 2003).
4. Male median lobe; (0) asymmetrical, (1) symmetrical (Figs 31, 34, 37, 40, 43, 45, 47, 49) (Char. 62, Miller 2001).
5. Apex of ventral sclerite; (0) various, (1) bifid (Figs 31, 34), (2) expanded and bilobate, spiny (Figs 42, 43, 45, 47).

***Female genitalia***

1. Female genital configuration; (0) Hydroporinae-type; (1) Dytiscinae-type (Char. 1, Miller 2001).
2. Accessory glands (or possibly other structures) on either side of base of common oviduct; (0) absent, (1) present (Figs 58, 59, 61–66) (Char. 32, Miller 2001).
3. Very large muscles surrounding vagina; (0) absent, (1) present (Char. 33, Miller 2001).
4. Series of short, stiff setae along medial margin of gonocoxosternite; (0) absent, (1) present (Figs 60, 62, 63) (Char. 14, Miller 2001).
5. Gonocoxae; (0) not fused, (1) fused along medial margin (Figs 58–66) (Char. 41, Burmeister 1976; Miller 2000).
6. Apicolateral setal pencil on gonocoxa; (0) absent, (1) present (Char. 44, Miller 2000).
7. Medial margin of gonocoxa; (0) not emarginate (Figs 60–66), (1) emarginate (Figs 58, 59).
8. Rami at apex of vagina; (0) smooth (Figs 58–63, 66), (1) laterally corrugated (Figs 64, 65).

**Larva**

***Head***

1. Anterior margin of clypeus; (0) evenly curved, (1) strongly excavated (Figs 67–71) (Char. 70, Miller 2003).
2. Rudimentary visual organ; (0) absent, (1) present. In larvae of Dytiscinae, anteromedially of the anterior row of stemmata there is a clear and rounded spot in the cuticle (Ferreira Jr 2000) called a “rudimentary visual organ” by Blunck (1917) and an “ocular spot” by Bertrand (1928).
3. Subdivision of the cephalic appendages (antenna, maxillary and labial palpi) into articles; (0) not subdivided, (1) subdivision of at least one segment into two articles, beginning with the second instar, (2) subdivision of at least one segment into three articles, beginning with the first instar (non-additive).
4. Antennomere I; (0) not subdivided, (1) subdivided into two articles.
5. Antennomeres II and III, subdivision; (0) not subdivided, (1) basal segment subdivided resulting in two unequal articles, beginning in the second instar, (2) basal segment subdivided into three articles, subequal in length, beginning with the first instar (non-additive).
6. Galea; (0) absent, (1) present.
7. Maxillary palpomere I (not including palpifer); (0) not subdivided, (1) basal segment subdivided, resulting in two unequal articles, beginning with the second instar, (2) basal segment subdivided into two subequal articles beginning with the first instar (non-additive).
8. Maxillary palpomere II; (0) not subdivided, (1) basal segment subdivided, resulting in two unequal articles beginning with the second instar, (2) basal segment subdivided into two subequal articles beginning with first instar (non-additive).
9. Maxillary palpomeres III; (0) not subdivided, (1) basal segment subdivided, resulting in two unequal articles, (2) basal segment subdivided into two subequal articles (non-additive).
10. Apicomedial margin of labial prementum; (0) unmodified, (1) with single rounded projection, (2) with single, elongate, spinous projection.
11. Labial palpomeres; (0) not secondarily subdivided, (1) secondarily subdivided.

***Abdomen***

1. Abdominal tergites I–VI; (0) large, covering dorsum of each segment (1) reduced to a small plate located anteriorly on dorsum of segment.
2. Venter of abdominal segment VII; (0) not sclerotized, (1) sclerotized.
3. Urogomphi; (0) reduced, (1) well developed.
4. Urogomphi; (0) without fringe of setae, (1) with fringe of setae along lateral margins only; (2) with fringe of setae along lateral and medial margins.

## Supplementary materials

Supplementary material 1 Character coding for phylogenetic analysis Data type docx https://binary.pensoft.net/file/963595This dataset is made available under the Open Database License (http://opendatacommons.org/licenses/odbl/1.0/). The Open Database License (ODbL) is a license agreement intended to allow users to freely share, modify, and use this Dataset while maintaining this same freedom for others, provided that the original source and author(s) are credited.Kelly B. Miller, Mariano C. Michat, Nelson Ferreira Jr
