# Supplementary material for: ﻿Reclassification of Cybistrinae Sharp, 1880 in the Neotropical Region (Coleoptera, Adephaga, Dytiscidae), with description of new taxa
Source: Zookeys. 2024 Jan 8;1188:125–68. doi: 10.3897/zookeys.1188.110081 (PMC10790577; doi:10.3897/zookeys.1188.110081)
Supplement: Supplementary material 1 — Character coding for phylogenetic analysis [file zookeys-1188-125_article-110081__-s001.docx]

**Supplementary Table 1.** Character coding for phylogenetic analysis. $ = 4,5. Characters marked with + were analyzed as additive.

|  | 0000000001 1111111112 2222222223 3333333334 4444444445 5555555  1234567890 1234567890 1234567890 1234567890 1234567890 1234567  + + |
| --- | --- |
| *Lancetes lanceolatus* | 0100100001 1001011000 0000001000 0000000001 0000000100 0000110 |
| *Lancetes varius* | 0100100001 1001011000 0000001000 0000000001 00-------- ------- |
| *Rhantus binotatus* | 0100110001 1001011000 0000001011 000--00001 0000000100 0000110 |
| *Meridiorhantus calidus* | 0101110101 1001011000 0000001011 000--00001 0000000100 0000110 |
| *Dytiscus marginalis* | 1001110100 1101112010 0000101011 0110100010 0001111111 1010112 |
| *Dytiscus verticalis* | 1001110100 1101112010 0000101011 0110100010 0001111111 1010112 |
| *Hyderodes shuckardi* | 10000-0000 0101012010 0000101010 0010100011 0001111111 1010111 |
| *Hydaticus cinctipennis* | 10010-0100 0111012000 0001001000 0010100011 00-------- ------- |
| *Notaticus fasciatus* | 10000-0000 0111012010 0001001000 0010100010 0001101100 1000110 |
| *Eretes australis* | 10000-0000 0111012000 0001001000 0010100011 0001101100 1200110 |
| *Acilius abbreviatus* | 10000-0000 0111012000 0001001000 0010100011 0001101100 1200110 |
| *Austrodytes plateni* | 0001120111 100100-000 1110001001 0010111010 00-------- ------- |
| *Sternhydrus atratus* | 0011120111 1001010000 1110000001 0010111010 0011212022 2111000 |
| *Onychohydrus scutellaris* | 0001120111 1001110000 1110000001 0010111010 0011212022 2111000 |
| *Spencerhydrus latecinctus* | 0011120111 101000-000 1110001001 0010111010 0011212022 2111000 |
| *Spencerhydrus pulchellus* | 0011120111 101000-000 1110001001 0010111010 0011212022 2111000 |
| *Metaxydytes carcharias* | 0001121011 1001010001 1110201001 0010111110 0011212022 2111000 |
| *Metaxydytes fraternus* | 0001121011 1001010001 1110201001 0010111110 0011212022 2111000 |
| *Metaxydytes laevigatus* | 0001121011 1001010001 1110201001 0010111110 00-------- ------- |
| *Metaxydytes marginithorax* | 0001121011 1001010001 1110201001 0010111110 00-------- ------- |
| *Paramegadytes glaucus* | 0000120011 1001010001 1110201001 0010111110 0011212022 2111000 |
| *Trifurcitus robustus* | 0001121111 1001010001 1110101101 0010111010 0111212022 2111100 |
| *Bifurcitus lherminieri* | 0001121111 1001010001 1110101101 0010111010 01-------- ------- |
| *Nilssondytes diversus* | 00111201?1 1001010001 1110301001 0010111010 00-------- ------- |
| *Cybister latus* | 00001210?1 1001010001 1110301001 1012???010 0011212022 2111000 |
| *Cybister parvus* | 00001210?1 1001010001 1110301001 1012?????? ??-------- ------- |
| *Cybister brevis* | 0000120011 1011010101 1110401001 1010111010 00-------- ------- |
| *Cybister immarginatus* | 0000120011 1011010101 1110401001 1010111010 00-------- ------- |
| *Cybister convexus* | 0000120011 1011010101 1110401001 1010111010 00-------- ------- |
| *Cybister posticus* | 0000120011 1011010101 1110401001 1010111010 00-------- ------- |
| *Cybister sugillatus* | 0000120011 1011010101 1110401001 1010111010 00-------- ------- |
| *Cybister vicinus* | 0000120011 1011010101 1110401001 1010111010 00-------- ------- |
| *Cybister burgeoni* | 0000120011 1011010101 1110401001 1010111010 00-------- ------- |
| *Cybister vulneratus* | 0000122011 1011010101 1110401001 1010111010 00-------- ------- |
| *Cybister marginicollis* | 0001120011 1011010101 1110401001 1010111010 00-------- ------- |
| *Cybister explanatus* | 0001122111 1011010101 1110$11001 1010111010 00-------- ------- |
| *Cybister fimbriolatus* | 0001122111 1011010101 1110$11001 1010111010 0011212022 2111000 |
| *Cybister japonicus* | 0001122111 101100-101 1111501001 1010111010 00-------- ------- |
| *Cybister lateralimarginalis* | 0001122111 101100-101 1111501001 1010111010 0011212022 2111000 |
| *Cybister cognatus* | 0001122111 1011010101 1110501001 1010111010 00-------- ------- |
| *Cybister guerini* | 0001122111 1011010101 1110501001 1010111010 00-------- ------- |
| *Cybister ventralis* | 0001122111 1011010101 1110501001 1010111010 00-------- ------- |
| *Cybister cardoni* | 0001120111 1011010101 1110501001 1010111010 00-------- ------- |
| *Cybister senegalensis* | 0001120111 1011010101 1110501001 1010111010 00-------- ------- |
| *Cybister tripunctatus* | 0001120111 1011010101 1110501001 1010111010 0011212022 2111000 |
| *Cybister cinctus* | 0001120111 1011010101 1110501001 1010111010 00-------- ------- |
| *Cybister gschwendtneri* | 0001120111 1011010101 1110501001 1010111010 00-------- ------- |
| *Cybister puncticollis* | 0001120111 1011010101 1110501001 1011111010 10-------- ------- |
| *Cybister festae* | 0001120111 1011010101 1110501001 1011111010 10-------- ------- |
